# VSIG4 Promotes Tumour‐Associated Macrophage M2 Polarization and Immune Escape in Colorectal Cancer via Fatty Acid Oxidation Pathway

**DOI:** 10.1002/ctm2.70340

**Published:** 2025-05-22

**Authors:** Jiafeng Liu, WenXin Zhang, Lu Chen, Xinhai Wang, Xiang Mao, Zimei Wu, Huanying Shi, Huijie Qi, Li Chen, Yuxin Huang, Jiyifan Li, Mingkang Zhong, Xiaojin Shi, Qunyi Li, Tianxiao Wang

**Affiliations:** ^1^ Department of Pharmacy, Huashan Hospital Fudan University Shanghai China; ^2^ Department of Surgery, Huashan Hospital Fudan University Shanghai China; ^3^ Department of Pharmacy, Shanghai Xuhui Central Hospital, Zhongshan‐Xuhui Hospital Fudan University Shanghai China

**Keywords:** colorectal cancer, immunotherapy, macrophages, metabolic reprogramming, VSIG4

## Abstract

**Background:**

V‐set and immunoglobulin domain containing 4 (VSIG4) is a B7‐family‐related protein almost exclusively expressed on macrophages. The difference in its expression mediates the dynamic transformation of the polarization state of macrophages, but the underlying mechanism is still unclear. We sought to reveal the correlation between VSIG4 and the polarization of tumour‐associated macrophages (TAMs) and the immune escape of tumour cells in colorectal cancer (CRC).

**Methods:**

THP‐1 monocyte‐derived macrophages expressing different levels of VSIG4 were used for in vitro investigations. In addition, the co‐culture system was used to verify the effect of tumour cells on the expression of VSIG4 in macrophages, and the effect of VSIG4 expression level on tumour cells in turn. Subcutaneous xenograft models evaluated the tumour growth inhibition efficacy of VSIG4 blockade as monotherapy and combined with immune checkpoint inhibitors (ICIs).

**Results:**

CRC cells secreted lactate to promote VSIG4 expression in macrophages. On the contrary, VSIG4 promoted macrophage M2 polarization and induced malignant progression of tumour cells by promoting M2 macrophage secretion of heparin‐bound epidermal growth factor. In vivo experiments confirmed that knockdown VSIG4 inhibited tumour growth and improved the efficacy of ICIs therapy. Mechanistically, lactate secreted by CRC cells promoted its expression by influencing the epigenetic modification of VSIG4 in macrophages. In addition, VSIG4 enhanced the fatty acid oxidation (FAO) of macrophages and upregulated PPAR‐γ expression by activating the JAK2/STAT3 pathway, which ultimately induced M2 polarization of macrophages. Downregulation of VSIG4 or blocking of FAO reversed the M2 polarization process of macrophages.

**Conclusions:**

Our findings provide a molecular basis for VSIG4 to influence TAMs polarization by regulating the reprogramming of FAO, suggesting that targeting VSIG4 in macrophages could enhance the ICIs efficacy and represent a new combination therapy strategy for immunotherapy of CRC.

**Key points:**

- Colorectal cancer cells secrete lactate to upregulate VSIG4 in macrophages via the H3K18la‐METTL14‐m6A axis.
- VSIG4 promotes fatty acid oxidation of macrophages and drives its M2‐type polarization.
- These VSIG4‐expressing M2 macrophages promote tumour progression and an immunosuppressive microenvironment.
- Inhibition of VSIG4 expression can synergistically enhance the therapeutic effect of anti‐PD‐1 antibody.

## Graphical abstract

1. Colorectal cancer cells secrete lactate to upregulate VSIG4 in macrophages via the H3K18la‐METTL14‐m6A axis.
2. VSIG4 promotes fatty acid oxidation of macrophages and drives its M2‐type polarization.
3. These VSIG4‐expressing M2 macrophages promote tumour progression and an immunosuppressive microenvironment.
4. Inhibition of VSIG4 expression can synergistically enhance the therapeutic effect of anti‐PD‐1 antibody.

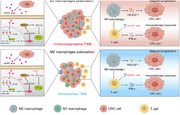

## 1 BACKGROUND

Colorectal cancer (CRC) ranks as the third most prevalent lethal malignancy globally and represents the second leading contributor to cancer mortality.^1^ Although CRC prevalence and mortality rates have continued to decline over the past decade, they have continued to increase among young adults. Current standard therapies (surgery/radiotherapy/chemotherapy) show constrained efficacy with significant adverse effects, driving demand for novel therapeutic strategies.^2^ Immunotherapeutic strategies targeting PD‐1/CTLA‐4 pathways have revolutionized CRC management. These immune checkpoint inhibitors enhance endogenous antitumour responses, demonstrating substantial clinical efficacy.^3^ However, due to differences in the patient's own tumour immune microenvironment (TIME), the majority of patients are insensitive to immunotherapy or develop resistance.

Mounting evidence highlights TIME's pivotal role in tumorigenesis and progression.^4^ This dynamic niche comprises immune/stromal cells, extracellular matrix, and secretory factors.^5^ Key immunosuppressive drivers include tumour‐associated macrophages (TAMs), bone marrow‐derived suppressor cells (MDSCs), and regulatory T cells (Tregs) within TIME.^6^ As dominant tumour‐infiltrating immune cells, macrophages exhibit M1/M2 polarization dynamics, a well‐established functional dichotomy (M1: classical activation; M2: alternative activation) with robust empirical support.^7^ M1 macrophages mainly express anti‐tumour effects. Conversely, M2 macrophages suppress pro‐inflammatory immune functions and promote angiogenesis and tumour progression by secreting IL‐10, TGF‐β, vascular endothelial growth factor and arginase‐1 (ARG1).^8^ TAMs typically display features characteristic of M2 macrophages.^9^, ^10^, ^11^ In addition, M2 macrophages disrupt anti‐tumour immunity and enhance tumorigenesis through multiple mechanisms. These include suppressing T cell function and metabolism by secreting immunosuppressive cytokines, thereby enabling the immune escape of tumour cells.^12^ Extensive research demonstrates TAMs' correlation with unfavourable outcomes in various cancer patients.^13^ Therefore, further investigation of the mechanisms of macrophage polarization and the interaction between macrophage polarization and CRC could help develop novel therapeutic strategies or assist in enhancing the efficacy of immune checkpoint blockade (ICB) therapy.

The metabolic reprogramming of macrophages assumes a pivotal role in their polarization.^14^ For example, glycolysis is required for the M1 polarization state of macrophages. Stimulation by lipopolysaccharide (LPS) has the capacity to enhance aerobic glycolysis in macrophages, thus driving M1‐dominant macrophage polarization.^15^ The activation of pyruvate kinase M2, a glycolytic enzyme, inhibits aerobic glycolysis, consequently diminishing LPS‐induced macrophage polarization towards the M1 phenotype.^16^ Conversely, M2 macrophages exhibit a greater reliance on oxidative phosphorylation, characterized by a higher number of mitochondria and elevated oxygen consumption rates.^17^ Fatty acid oxidation (FAO) is thought to be an important energy source for the polarization of macrophages towards the M2 phenotype.^18^ When macrophages are stimulated with interleukin‐4 (IL‐4), there is an increase in fatty acid uptake and oxidation, along with an augmentation in mitochondrial biosynthesis.^19^ Inhibiting FAO in TAMs promotes anti‐tumour polarization of TAMs and impedes tumour growth.^20^ Accumulating research has indicated that FAO in TAMs is dependent on the activation and induction of peroxisome proliferator‐activated receptor‐γ (PPARγ), which is also indispensable for the tumour polarization of TAMs.^21^ However, the mechanistic insight accounted for the fatty acid metabolism reprogramming of TAMs in CRC remains to be clarified.

V‐set immunoglobulin‐domain‐containing 4 (VSIG4), a member of the immunoglobulin superfamily, also known as CRIg or Z39Ig, primarily expressed on tissue macrophages in most digestive organs (including liver, pancreas and intestines).^22^, ^23^ Emerging as a novel immune checkpoint, VSIG4 suppresses IL‐2 synthesis and T cell expansion, highlighting its anti‐inflammatory regulatory function.^23^ VSIG4 also suppresses macrophage M1‐type differentiation by restricting mitochondrial pyruvate metabolism.^24^ Emerging evidence links elevated VSIG4 expression to poorer clinical outcomes across multiple solid tumours (e.g., glioma, gastric, breast, lung, pancreatic), positioning this checkpoint protein as a crucial modulator of tumour progression.^25^, ^26^, ^27^, ^28^ However, the involvement of VSIG4 in CRC pathogenesis and therapeutic approaches remains to be elucidated.

In this study, we discovered that CRC cell‐derived lactate triggers macrophage histone lactylation, which drives METTL14 upregulation and subsequent m6A modification. This cascade ultimately results in an increased expression of VSIG4 in macrophages by stabilizing the VSIG4 mRNA. Furthermore, VSIG4 upregulates macrophage PPAR‐γ expression and FAO via the JAK2/STAT3 signalling pathway, thereby fostering the polarization of macrophages towards the M2 subtype. VSIG4 inhibition demonstrated dual efficacy in suppressing oncogenesis and reprogramming the tumour immune microenvironment (TIME), with synergistic enhancement of PD‐1 blockade.

## 2 MATERIALS AND METHODS

### 2.1 Data acquisition and preprocessing

TCGA‐derived datasets encompassed 616 CRC cases and 51 matched normal tissues, with clinicopathological annotations (age, sex, survival, lymphatic invasion, histological grade I‐IV, TNM staging). Differentially expressed genes (DEGs) in CRC datasets were identified with thresholds of |logFC| > 1.5 and *p* < .05. The data set of GSE14333 is obtained from the GEO database and normalized with the R package “limma”. The CRC samples with available clinical characteristics from the TCGA database were treated as the training dataset and the CRC samples from the GSE14333 were treated as the validation dataset. The immune scores of each tumour sample from the TCGA database were calculated by the R package “ESTIMATE” based on R language software (version 4.2.2).^29^

### 2.2 Patients and specimens

CRC specimens and matched adjacent normal tissues (≥1 cm from tumour margins, microscopy confirmed tumour‐free) were procured from Huashan Hospital (2017‐2023). VSIG4/CD8A expression quantification employed real‐time PCR and IHC, with prognostic correlations assessed. This study received Ethics Committee approval with written consent documentation from all enrolled subjects.

### 2.3 Cell culture

Human CRC lines (SW620, HT29, LOVO), murine MC38, and THP‐1 monocytes were sourced from the Chinese Academy of Sciences Cell Bank. Culture media comprised RPMI‐1640 (HT29/THP‐1/MC38), L‐15 (SW620), and F12K (LOVO) from Hyclone, supplemented with 10%FBS (Yeasen Biotechnology Co., Ltd) and 100 U/mL antibiotics (Wisent), maintained in a humidified incubator at 37°C with 5% CO_2_.

### 2.4 In vivo treatments

All methodological and ethical details of in vivo studies conducted in this study are consistent with those described in our previous publications.^30^ To establish immunocompetent murine models, 1×10⁷ CRC cells were subcutaneously inoculated into C57BL/6 mice. Tumour progression was assessed at 4‐day intervals post‐implantation. When tumours attained 100 mm^3^ volume, intratumoral delivery of VSIG4 siRNA (2.5 nmol/20 g) was implemented to inhibit VSIG4 expression, with parallel administration of scrambled siRNA in control animals. In select experimental groups, clodronate‐loaded liposomes (200 µL/animal, q5d) were administered via intraperitoneal delivery to achieve TAMs depletion. CD8^+^ T cell elimination was accomplished by pretreating animals with 100 µg anti‐CD8 mAb (BioXCell, #BE0061) or matched IgG control through intraperitoneal administration 24 h prior to tumour cell implantation. The PD‐1 blockade regimen involved systemic delivery of anti‐mouse PD‐1 mAb (100 µg/mouse) or isotype control (BioXCell) via intraperitoneal route on days 4, 8, 12, and 16 post‐tumour challenge. Tumours were measured every 4 days until day 24 of euthanasia. Volumes were calculated using *V* = .5 × *l* × *w^2^*. Excised tissues were then weighed.

### 2.5 Statistical analysis

Statistical analyses utilized GraphPad Prism 7.0 and SPSS 16.0. In vitro group comparisons employed one‐way ANOVA with Dunnett's post hoc. Animal data employed unpaired *t*‐tests. Pre‐analysis validation included Kolmogorov‐Smirnov (normality) and Levene's (variance) tests. All data met assumptions. Results are expressed as mean ± SD. Significance: *p* < .05.

## 3 RESULTS

### 3.1 VSIG4 expression correlates with immune infiltration levels and prognostic significance in CRC

**FIGURE 1:**
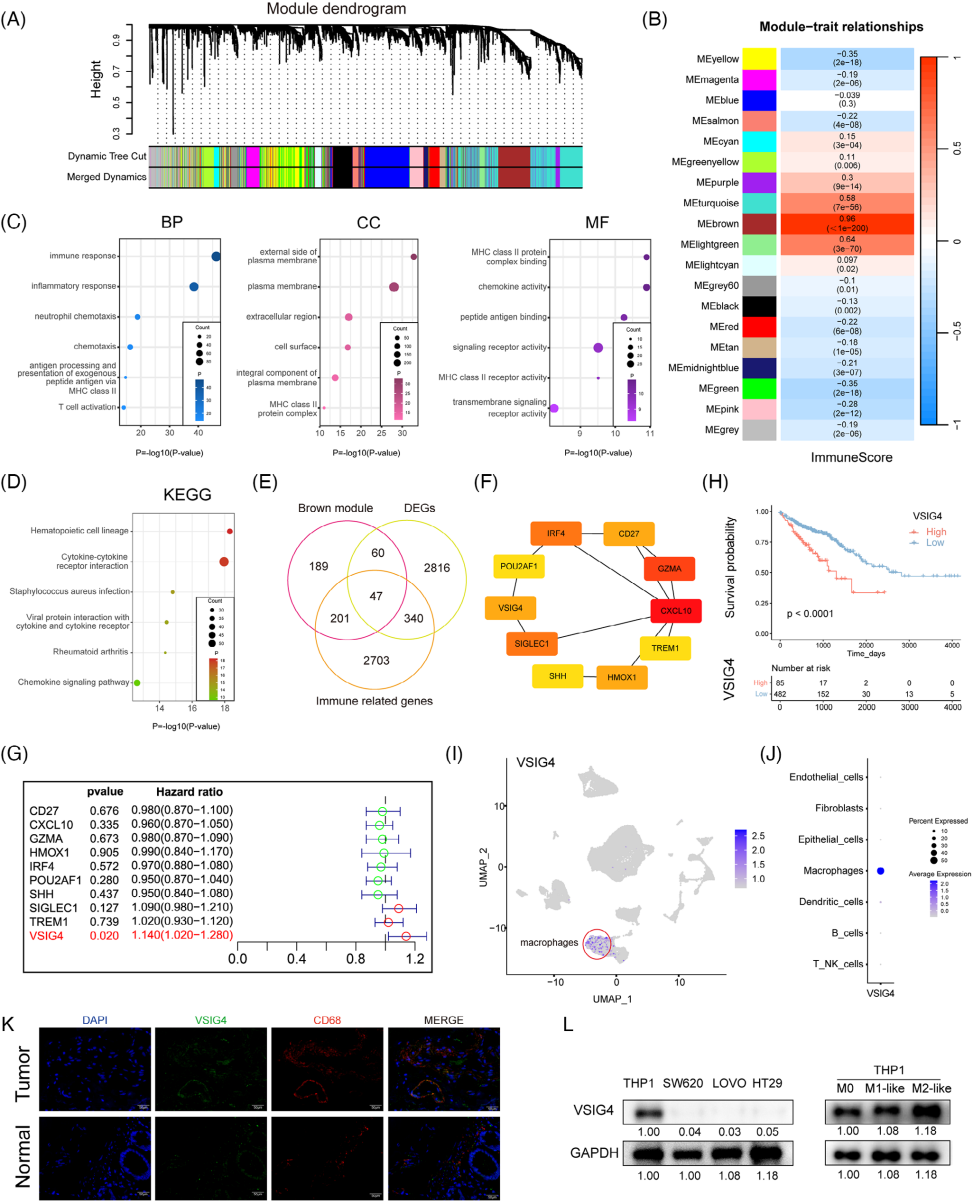
VSIG4 expression correlates with immune infiltration levels and prognostic significance in CRC. (A) In the TCGA‐CRC cohort, the hierarchical clustering tree was constructed by the dynamic hybrid cutting method, and a total of 19 co‐expression models were generated. (B) Correlation coefficients between ImmuneScores and co‐expression modules. The brown module had the strongest correlation with ImmuneScore (COR = .97; *p* < .001). GO (C) and KEGG (D) analyses showed the relevant biological processes (BP), cellular components (CC), molecular functions (MF) and pathways. (E) Venn diagram of DEGs, brown modules and immune‐related genes. (F) The top 10 Hub genes were identified using the CytoHubba plugin of Cytoscape software. (G) Univariate Cox proportional risk regression models were used to analyze the association between the 10 Hub genes and OS in the TCGA‐CRC cohort. (H) Survival analysis showing the relationship between VSIG4 expression level and the OS in TCGA. Single‐cell sequencing analysis showed that in 10 pairs of primary colorectal cancer samples and matched normal mucosal samples, VSIG4 was predominantly expressed by macrophages (UMAP (I) and dot plot (J)). (K) Immunofluorescence staining showed colocalization of VSIG4 and the macrophage marker CD68. (L)Western blot analysis was used to detect the protein expression of VSIG4 in CRC cells, THP‐1 monocytes and THP‐1 monocytes after induction of different polarization.

We downloaded the gene expression profiles of 616 CRC tissues and 51 adjacent normal tissues from the TCGA database, and evaluated ImmuneScore for each patient by “ESTIMATE”. The WGCNA co‐expression network was constructed, and we found that the brown module exhibited maximal co‐expression alignment with patient ImmuneScore (*R* = .96) (Figure 1A,B). Therefore, the brown module with the highest positive correlation coefficient was selected for enrichment analysis to verify the accuracy of WGCNA analysis (Figure 1C,D). To find out which genes play a major role in cancer, we compared DEGs between CRC tissue and normal tissue, with a FoldChang greater than 1.5 defined as a difference, identifying 3263 genes (Figure S1A). Then, the intersection of 3263 DEGs, 3291 immune‐related genes, and 496 brown modules genes included 47 key genes (Figure 1E). A protein–protein interaction network (PPI) was constructed for these 47 key genes by the STRING tool and then uploading the PPI network to Cytoscape software (Figure S1B). Ten genes were identified as hub genes by the CytoHubba plugin (Figure 1F). Ultimately, Univariate Cox regression identified VSIG4 as the sole hub gene with prognostic relevance in CRC (HR = 1.14, 95%CI 1.02–1.28, *p* = .02) (Figure 1G). Consequently, VSIG4 was identified as the target gene for subsequent analysis and validation.

Subsequently, Kaplan–Meier analysis was used to reveal the prognostic correlation of VSIG4 expression in CRC cohorts, and the result showed that high VSIG4 expression was associated with poor prognosis for CRC patients (Figure 1H). Subsequently, Functional enrichment analyses (GO/KEGG) revealed VSIG4's predominant involvement in immune‐inflammatory pathway regulation within CRC (Figure S1C–F). To delve deeper into the interplay between VSIG4 and immune function, we employed GSVA to determine the enrichment score of immune processes. Correlation analysis between enrichment score and VSIG4 expression demonstrated that VSIG4 was significantly positively correlated with immune function (Figure S2A,B). What's more, we quantified the relative abundance of immune cell infiltration in CRC patients' TIME using CIBERSORT and ssGSEA, as shown in Figure S2C,D, the proportion of tumour‐infiltrating immune cells varied significantly between the high and low expression subgroups of VSIG4. These findings above indicate that VSIG4 expression is significantly correlated with immune infiltration and prognosis significance in CRC.

### 3.2 VSIG4 is selectively upregulated in M2‐like macrophages in colorectal cancer tumour microenvironment

In elucidating the cellular provenance of VSIG4 in CRC tissues, we analyzed single‐cell RNA sequencing data obtained from 10 pairs of primary CRC samples and matched normal mucosal samples from the GSE132465 database. After quality control processes, all the cells were divided into 25 clusters, which were further stratified into seven discrete categories based on their marker genes (Figure S3A–C). Among them, cluster 6 was identified as macrophages (Figure S3D). Our investigation revealed that VSIG4 predominantly manifests itself in the macrophage population, with negligible presence across other cellular categories (Figure 1I,J). This finding was further substantiated by an examination of the Single Cell Center for Tumour Immunity database, wherein the VSIG4 gene exhibited specific expression on macrophages across diverse tumour types (Figure S3E). Furthermore, immunofluorescence staining revealed colocalization of VSIG4 with CD68, a recognized marker for macrophages, alongside a marked elevation of VSIG4 expression in tumour tissues (Figure 1K). To verify the above findings, Western blot experiments were performed in macrophage cell lines and CRC cell lines and found that VSIG4 was expressed in macrophages but hardly expressed in CRC cells (Figure 1L). Furthermore, correlation analysis revealed a positive association between VSIG4 and CD68, as well as CD163, the hallmark genes of M2 macrophages (Figure S3F). Notably, M2‐polarized macrophages exhibited marked VSIG4 upregulation compared with M0/M1 phenotypes (Figure 1L). Additionally, using the GEPIA 2021 online tool, we found a significant upregulation of VSIG4 gene expression in M2 macrophages across various tumour types, including CRC (Figure S3G). In summary, these observations suggest that VSIG4 is predominantly expressed on macrophages, and increased in expression on M2 macrophages.

### 3.3 VSIG4 promotes M2 polarization of macrophages and induces malignant progression of CRC cells through HB‐EGF

In light of the critical role macrophages play as constituents of immune cells infiltrating tumours,^7^ our conjecture stands that VSIG4 triggers immunosuppression through the facilitation of macrophage polarization and recruitment. Initially, THP‐1‐derived macrophages (M0) were polarized into M1/M2 phenotypes using LPS/IFN‐γ or IL‐4/IL‐13 polarizing stimuli (Figure S4A). Furthermore, mRNA expressions of CCR7, CD80, and NOS2 were notably up‐regulated in M1 macrophages. In contrast, M2 macrophages displayed a significant increase in mRNA expression of ARG1, CD163, and CLEC7A (Figure S4B). Therefore, these results collectively imply successful polarization of THP‐1 monocytes into M1 and M2‐polarized macrophages.

**FIGURE 2:**
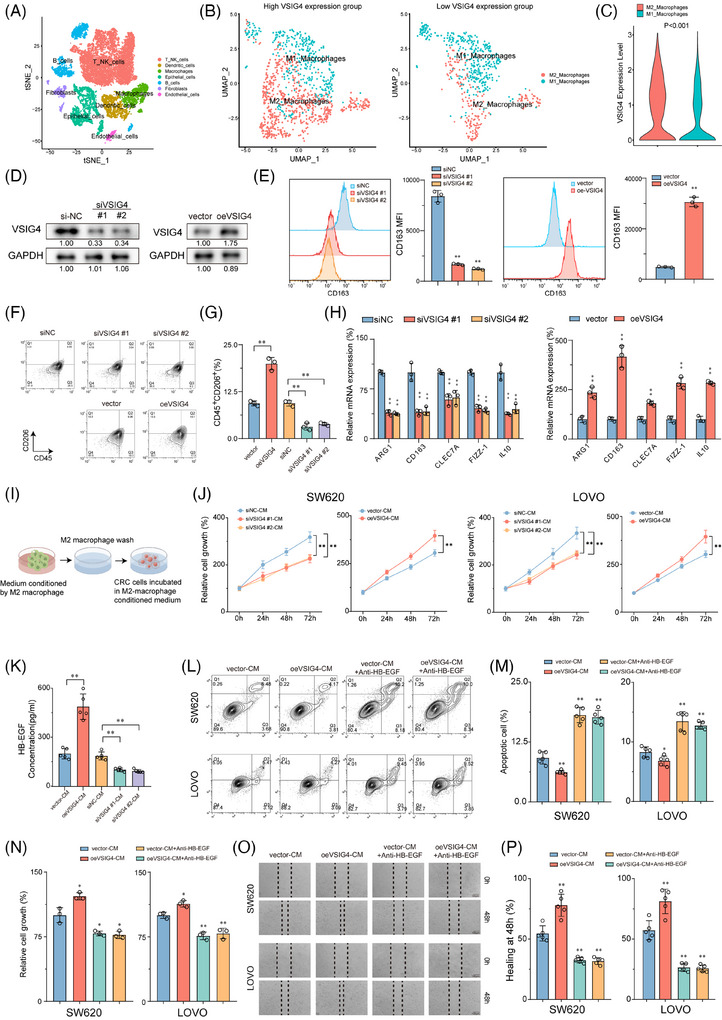
VSIG4 promotes M2 polarization of macrophages and induces malignant progression of CRC cells through HB‐EGF. (A) T‐distributed Stochastic Neighbor Embedding (t‐SNE) plot from 10 CRC patients. (B) t‐SNE plot of macrophage categories with high VSIG4 expression group (left) and low expression group (right). (C) Expression of VSIG4 in M1 macrophages and M2 macrophages. (D) The protein expression of VSIG4 in macrophages was detected by Western blot analysis. (E) The distribution of CD163^+^ macrophages was detected by flow cytometry (FCM). (F) and (G) The distribution of CD206^+^CD45^+^ macrophages were detected by FCM. (H) The mRNA levels of marker genes in M2 macrophages were detected by qRT‐PCR. (I) Schematic diagram of co‐culture of M2 macrophage‐derived CM with SW720 and LOVO. (J) The effect of M2 macrophage CM on CRC cell viability was determined by CCK8. (K) The content of HB‐EGF in M2 macrophage CM of each group was detected by ELISA. (L) and (M) FCM was used to observe the effect of VSIG4 on the apoptosis of CRC cells by regulating the secretion of HB‐EGF in macrophages. (N) CCK8 assay was used to observe the effect of VSIG4 on the viability of CRC cells by regulating the secretion of HB‐EGF in macrophages. (O, P) wound scratch assay was performed to observe the effect of VSIG4 on the invasion and migration of CRC cells by regulating the secretion of HB‐EGF in macrophages. *p < .05, **p < .01.

We then investigated the effect of VSIG4 on macrophage polarization. Ten primary CRC samples from the above single‐cell RNA sequencing data were selected for the following analysis (Figure 2A). Based on the expression level of VSIG4, ten samples were divided into two groups (Figure S4C). The macrophage categories were further classified according to the marker genes of M1 and M2 macrophages (Figure 2B). We found that the expression level of VSIG4 in M2 macrophages was higher than that in M1 macrophages (Figure 2C). Next, we knocked down VSIG4 expression in THP‐1 monocytes (siVSIG4) with VSIG4‐targeting siRNA sequences or constructed stable VSIG4‐overexpressing THP‐1 monocytes (oeVSIG4) with lentiviruses. As shown in Figure 2D, the expression of VSIG4 protein in THP‐1 monocytes was significantly decreased or increased, respectively. After induction of THP‐1 monocytes into M2 macrophages, the expression of CD163 and CD206 in M2‐polarized THP‐1 monocytes treated with small interfering RNA (siRNA) was significantly downregulated. In contrast, CD163 and CD206 expression was significantly upregulated in M2‐polarized THP‐1 monocytes after VSIG4 overexpression treatment (Figure 2E–G). To further verify the effect of VSIG4 expression levels on the polarization of macrophages, qRT‐PCR analysis measured mRNA levels of characteristic surface markers. The siVSIG4 group exhibited significantly lower expression of M2 macrophage markers (ARG1, CD163, CLEC7A, FIZZ‐1, IL‐10) compared with the control siRNA (siNC) group. Conversely, the oeVSIG4 group exhibited markedly increased M2 macrophage marker expression (Figure 2H). In addition, following 72 h IL‐4/IL‐13 stimulation of PMA‐activated THP‐1 monocytes, ELISA revealed reduced IL‐10 and TGF‐β concentrations in the siVSIG4 group (Figure S4D). Strikingly, VSIG4 expression levels exerted no effect on the M1 polarization of macrophages (Figure S4E). These results indicate that VSIG4 regulates the M2‐type polarization of macrophages.

M2 macrophages are a recognized subtype renowned for facilitating tumour migration, invasion, metastasis, and angiogenesis.^31^ Given the pivotal role of VSIG4 in M2 macrophage polarization, we embarked on an exploration to discern whether VSIG4‐induced M2 macrophages promoted CRC progression. Conditioned medium (CM) derived from differentially pretreated macrophages was utilized for subsequent co‐culture assays with CRC cell lines SW620 and LOVO (Figure 2I). The results showed that siVSIG4 knock‐downed M2 macrophages exhibited lower capacity in promoting tumour growth than siNC‐treated normal M2 macrophages, whereas oeVSIG4 M2 macrophages significantly enhanced the proliferation of CRC cells (Figure 2J). Given that M2 macrophages are known to induce epithelial‐mesenchymal transformation (EMT) in cancer cells, we explored the correlation between VSIG4 and several biomarkers associated with EMT processes (MMP9, SNAI1, SNAI2, VIM, TWIST1, TWIST2, CDH1, CDH2) in CRC through the TIMER2.0 website. Intriguingly, the results pointed to a significant association between VSIG4 and these key genes (Figure S5A). qRT‐PCR analysis further demonstrated that CM from oeVSIG4 M2 macrophages markedly upregulated the expression of EMT‐associated genes in CRC cells, providing mechanistic support for its role in promoting EMT progression (Figure S5B). Previous studies have demonstrated that M2 macrophages promote cancer cell growth in vitro by releasing growth factors such as heparin‐bound epidermal growth factor (HB‐EGF).^32^ Hence, we initially examined the production of HB‐EGF in M2 macrophage CM. The results showed that compared with the control group, the level of HB‐EGF in oeVSIG4 M2 macrophage CM was significantly increased, while the HB‐EGF content in siVSIG4 M2 macrophage CM was significantly decreased (Figure 2K). Furthermore, M2 macrophage CM was found to promote the proliferation and invasion and inhibit the apoptosis of CRC cells, with oeVSIG4 M2 macrophage CM further augmenting this effect. However, this effect of promoting the malignant progression of CRC was inhibited after anti‐HB‐EGF antibody treatment (Figure 2L–P; Figure S5C). In addition, knockdown VSIG4 significantly inhibited the invasion of CRC and promoted its apoptosis (Figure S5D). Taken together, these results suggest that VSIG4 promotes M2 polarization of macrophages and induces malignant progression of CRC cells through HB‐EGF.

### 3.4 Targeted reduction of VSIG4 expression inhibits tumour growth in vivo by regulating macrophage and CD8+ T cell function

**FIGURE 3:**
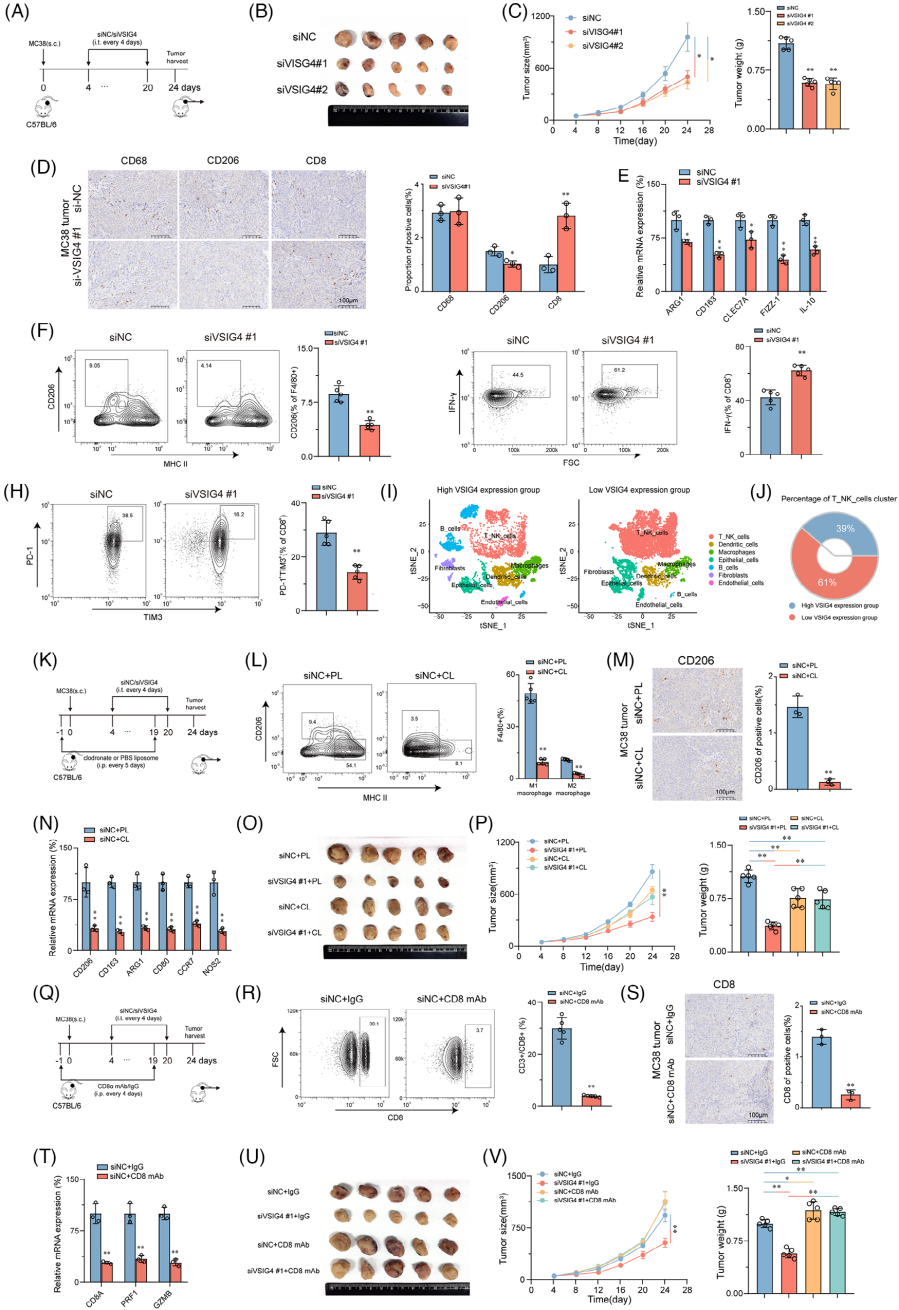
Targeted reduction of VSIG4 expression inhibits tumour growth in vivo by regulating macrophage and CD8^+^ T cell function. (A‐H) MC38 cells (5×10^5^) were subcutaneously (s.c.) transplanted into C57BL/7 mice, treated with isotype control or in vivo optimized small interfering RNA (siVSIG4) (*n* = 5). (A) Schematic diagram of the treatment plan. (B) Representative images of MC38 tumours. (C) The growth of MC38 tumours. (D) IHC staining of CD206, CD68 and CD8 specific antibodies. Scale, 100 µm. Quantified data was shown on the right. (E) The mRNA levels of marker genes in M2 macrophages were detected by qRT‐PCR. (F) The expression of tumour‐infiltrating F4/80^+^CD206^+^ TAMs in MC38 tumour‐bearing C57BL/7 mice was detected by FCM. (G, H) The specific molecular expression of tumour‐infiltrating CD8^+^ T cells in MC38 tumour‐bearing C57BL/7 mice was detected by FCM. (I) t‐SNE plots show the proportion of seven types of cells in the group with high VSIG4 expression (left) and the group with low VSIG4 expression (right). (J) Doughnut chart showing the T cell contents of VSIG4 high expression group and VSIG4 low expression group. (K–P) C57BL/7 mice were inoculated with MC38 cells and treated with siNC+PBS liposomes (PL), siVSIG4+PL, siNC+clodronate liposomes (CL) or siVSIG4+CL (*n* = 5), respectively (*n* = 5). (K)Schematic diagram of the treatment plan. (L) The distribution of tumour‐infiltrating CD206^+^ TAMs and MHC II+TAMs in MC38 tumour‐bearing C57BL/7 mice was detected by FCM. (M) CD206‐specific antibody immunohistochemical staining was used to detect the infiltration of CD206^+^ macrophages in subcutaneous transplanted tumours. Scale, 100 µm. (N) The mRNA levels of marker genes in M1 macrophages and marker genes in M2 macrophages were detected by qRT‐PCR. (O) Representative images of MC38 tumours. (P) The growth of MC38 tumours. (Q‐V) C57BL/7 mice were inoculated with MC38 cells and treated with siNC+IgG antibody, siVSIG4+IgG antibody, siNC+CD8 antibody or siVSIG4+CD8 antibody, respectively (*n* = 5). (Q) Schematic diagram of the treatment plan. (R) FCM was used to detect the distribution of tumour‐infiltrating CD8^+^ T cells in MC38 tumour‐bearing C57BL/7 mice. (S) CD8‐specific antibody immunohistochemical staining was employed to detect infiltration of CD8^+^ T cells in subcutaneous transplanted tumours. Scale, 100 µm. (T) The mRNA levels of CD8A, PRF1 and GZMB were detected by qRT‐PCR. (U) Representative images of MC38 tumours. (V) The growth of MC38 tumours. *p < .05, **p < .01.

**FIGURE 4:**
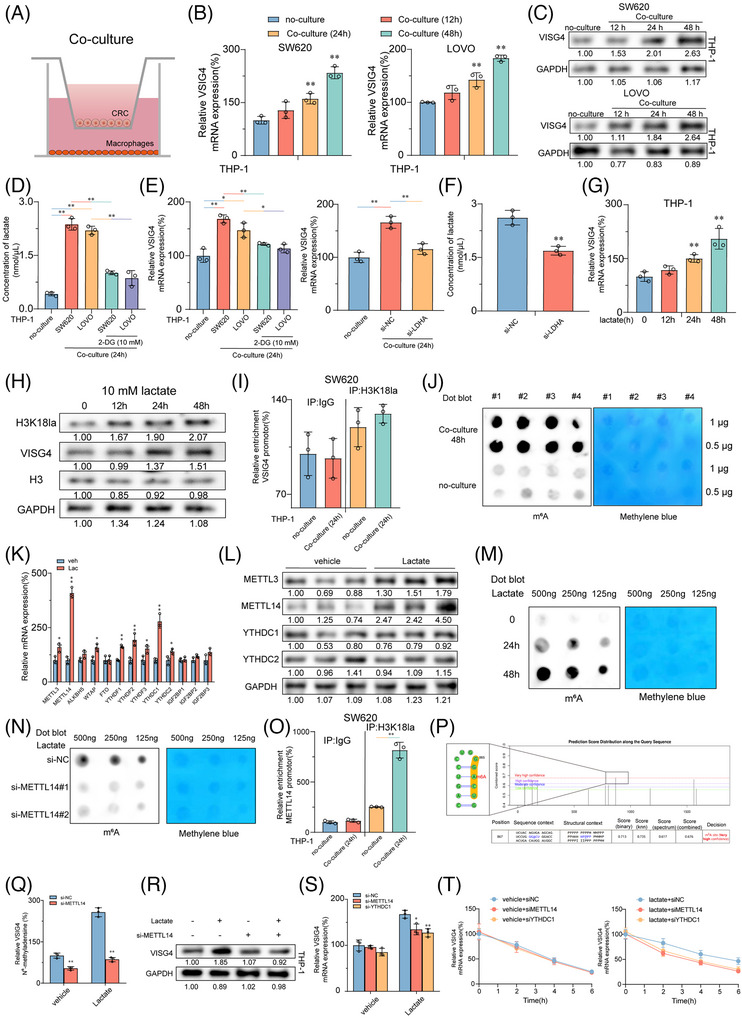
CRC cells secrete lactate to enhance the expression of VSIG4 in macrophages via the H3K18la‐METTL14‐m6A axis (A) Schematic diagram showing the Transwell co‐culture system of macrophages and CRC cells in vitro. (B) qRT‐PCR detected VSIG4 mRNA levels in PMA‐activated THP‐1 cells cocultured with SW620 (left) or LOVO (right) for 12, 24, and 48 h. (C) Western blot analysis was used to detect the expression of VSIG4 protein in PMA‐activated THP‐1 cells cocultured for 12, 24 and 48 h. (D) Lactate concentrations in the medium of PMA‐activated THP 1 cells cocultured with CRC cells for 24 h or in the control group were quantified by lactic acid assay kit with or without 2‐DG (10 mM) treatment. (E) qRT‐PCR was used to detect VSIG4 mRNA levels in PMA‐activated THP‐1 cells cocultured with CRC cells for 24 h with or without 2‐DG (10 mM) treatment (left); VSIG4 mRNA levels in PMA‐activated THP‐1 cells co‐cultured with si‐LDHA or si‐NC‐treated CRC cells were detected by qRT‐PCR (right). (F) Lactate concentration in the medium of si‐LDHA or si‐NC treated CRC cells was quantified by a lactic acid assay kit. (G) The mRNA levels of VSIG4 in PMA‐activated THP‐1 cells treated with lactic acid (10 mM) for 12, 24, and 48 h were detected by qRT‐PCR. (H) The protein expression of H3K18la and VSIG4 in PMA‐activated THP‐1 cells treated with lactic acid (10 mM) for 12, 24, and 48 h was detected by Western blot analysis. (I) PMA‐activated THP‐1 cells were co‐cultured with SW620 cells for 24 h, and the relative occupancy rates of H3K18la with the VSIG4 promoter were determined by ChIP‐qPCR. (J) The m6A abundance of total RNA in PMA‐activated THP‐1 cells co‐cultured with CRC cells for 48 h or in the control group was determined by Dot blot. (K) The mRNA levels of METTL3, METTL14, ALKBH5, WTAP, ALKBH5, FTO, YTHDF‐1, YTHDF‐2, YTHDF‐3, YTHDC‐1, YTHDC in PMA‐activated THP‐1 cells treated with lactate (10 mM) were detected by qRT‐PCR. (L) The protein expression of METTL3, METTL14, YTHDC1, and YTHDC2 in PMA‐activated THP‐1 cells treated with lactic acid (10 mM) was detected by Western blot. (M) Dot blot analysis showed that the m6A level of PMA‐activated THP‐1 cells changed with time after different concentrations of lactate treatment. (N) Dot blot analysis showed the m6A abundance of total RNA in PMA‐activated THP‐1 cells treated with si‐NC, si‐METTL14#1, or si‐METTL14# 2. (O) H3K18la enrichment at the METTL14 promoter in PMA‐activated THP‐1 cells stimulated by lactate or PBS for 12 h was analyzed by ChIP‐qPCR. (P) The m6A site of VSIG4 mRNA was predicted by SRAMP. (Q) The levels of m6A in VSIG4 in si‐NC or Si‐MettL14‐treated PMA‐activated THP‐1 cells in the lactate‐treated and control groups were measured by MeRIP‐qPCR. (R) The protein expression of VSIG4 in PMA‐activated THP‐1 cells treated with si‐NC or si‐METTL14 was detected by Western blot after 6 h of lactate treatment. (S) The mRNA levels of VSIG4 in PMA‐activated THP‐1 cells treated with si‐NC, si‐METTL14 or si‐YTHDC1 were detected by RT‐qPCR after 6 h of lactate treatment. (T) The mRNA levels of VSIG4 in PMA‐activated THP‐1 cells in si‐NC, si‐METTL14, or Si‐YTHDC1‐treated or control groups after 0, 2, 4, and 6 h of actinomycin D treatment. *p < .05, **p < .01.

To further investigate whether VSIG4 regulates macrophage polarization to influence tumour growth in vivo, we administered either in vivo‐optimized siRNA targeting the VSIG4 gene (siVSIG4) or siNC to immunoactive mice that had been inoculated with MC38 cells (Figure 3A). Results showed that optimized siRNA in vivo significantly reduced VSIG4 expression (Figure S6A,B) and tumour growth (Figure 3B,C). Immunohistochemical (IHC) and qRT‐PCR analyses of tumour tissues showed that M2 TAMs were significantly reduced in siVSIG4‐treated mice (Figure 3D,E). This view was further confirmed by FACS analysis (Figure 3F; Figure S6C). Furthermore, siVSIG4 treatment significantly enhanced the infiltration of CD8^+^ T cells within the tumour (Figure 3D). Notably, we observed significant increases in the activity (IFN‐γ) of tumour‐infiltrating CD8^+^ T cells in the siVSIG4 group (Figure 3G), while PD‐1 and TIM‐3 (T cell exhaustion markers) exhibited marked downregulation (Figure 3H). In addition, based on the expression levels of VSIG4 illustrated in Figure 4C, ten primary CRC samples were divided into two groups for single‐cell sequencing analysis, and seven cell types were defined according to the marker genes of different cell types (Figure S6D). The findings revealed a significant increase in the infiltration of T cells and NK cells in patients exhibiting low VSIG4 expression (Figure 3I,J). Next, we employed clodronate liposomes to deplete macrophages in mice to verify whether siVSIG4‐mediated inhibition of CRC growth was macrophage‐dependent (Figure 3K). The results showed that macrophages were significantly depleted after treatment with clodronate liposomes (Figure 3L–N). Moreover, Macrophage exhaustion notably reversed siVSIG4‐mediated suppression of CRC progression (Figure 3O,P). Additionally, to explore CD8^+^ T cell‐dependent mechanisms in VSIG4 inhibition‐mediated tumour control, in vivo CD8^+^ T cell depletion was achieved through antibody administration (Figure 3Q). We found that the density and function of CD8^+^ T cells were significantly reduced after treatment with anti‐CD8 antibody (Figure 3R–T). And inhibition of tumour growth in siVSIG4‐treated mice was reversed by depleting CD8^+^ T cells with anti‐CD8 antibodies (Figure 3U,V). Taken together, the above experiments confirmed that in vivo inhibition of macrophage VSIG4 delays the progression of CRC and that this tumour inhibition is dependent on the presence of macrophages and CD8^+^ T cells.

### 3.5 CRC cells secrete lactate to enhance the expression of VSIG4 in macrophages via the H3K18la‐METTL14‐m6A axis

To explore the underlying mechanism of increased VSIG4 expression in macrophages in the TME, we constructed a co‐culture system of CRC cells with PMA‐activated THP‐1 monocytes (Figure 4A). The results indicated that when PMA‐activated THP‐1 monocytes were co‐cultured with CRC cells, mRNA and protein levels of VSIG4 were significantly increased (Figures 4B,C). Furthermore, macrophages exhibited a notable polarization towards the M2 phenotype (Figure S7A). The “Warbug” effect is one of the critical characteristics of tumorigenesis.^33^ We found a significant increase in lactate content in the co‐culture system, which was significantly alleviated by treatment with the glycolysis inhibitor (2‐DG) (Figure 4D). Notably, mRNA levels of VSIG4 were significantly suppressed following 2‐DG treatment. In addition, LDHA knockdown similarly inhibited the expression of VSIG4 in the co‐culture system (Figure 4E,F). In contrast, the addition of exogenous lactate to the culture medium of PMA‐activated THP‐1 monocytes significantly promoted VSIG4 expression, which gradually increased over time (Figure 4G). Histone lactylation is an epigenetic modification that directly stimulates gene transcription in chromatin.^34^, ^35^ We hypothesized that histone lactylation may serve as a potential mechanism through which lactate promotes VSIG4 expression. With the extension of lactate treatment time, H3K18 lactation (H3K18la) and VSIG4 levels gradually increased, demonstrating a correlation between them (Figure 4H). However, chromatin immunoprecipitation (ChIP) analysis revealed that H3K18la was not significantly enriched in the promoter region of the VSIG4 gene, suggesting that histone lactylation did not directly influence the expression of the VSIG4 gene (Figure 4I).

N6‐methyladenosine (m6A), a fundamental epigenetic modification, significantly mediates tumorigenesis and malignant progression through its dynamic regulatory functions.^36^ To elucidate the alterations in RNA m6A modification within macrophages, we quantified macrophage m6A‐RNA methylation levels using Dot Blot analysis in co‐cultured and control groups. Our findings revealed that tumour cells markedly induced the upregulation of m6A in macrophages (Figure 4J). Next, we investigated the expression profiles of key m6A methyltransferases and m6A reader proteins, which are associated with increased m6A RNA. QRT‐PCR demonstrated that the methyltransferase METTL14 and the reader protein YTHDC1 exhibited the most significant upregulation (Figure 4K). This observation was further validated by Western blot analysis (Figure 4L). To ascertain whether the upregulation of m6A in macrophages is mediated by lactate released from tumour cells, we employed Dot Blot to monitor changes in m6A levels in lactate‐stimulated macrophages. A temporal elevation of m6A methylation was observed in lactate‐stimulated macrophages, with methylation intensity correlating positively with treatment duration (Figure 4M). Furthermore, we downregulated METTL14 expression through siRNA transfection (Figure S7B). Results from Dot blot analysis illustrated that the knockdown of METTL14 inhibited the lactate‐induced upregulation of m6A modification (Figure 4N), suggesting that METTL14 serves as a critical regulator of lactate‐mediated m6A modification. Consequently, we assumed that lactate‐triggered METTL14 overexpression was contingent upon the enhancement of H3K18la. ChIP‐qPCR was employed to determine the enrichment of H3K18la at the METTL14 sequence. The findings revealed that lactate stimulation significantly upregulated H3K18la enrichment on METTL14 in PMA‐activated THP‐1 monocytes in vitro (Figure 4O).

Next, we used the SRAMP platform to forecast potential m6A modification sites on VSIG4 and designed primers targeting those sites with the highest scores (Figure 4P). ME‐RIP analysis revealed that lactate significantly enhanced the abundance of m6A on VSIG4. However, the knockdown of METTL13 in PMA‐activated THP‐1 monocytes with si‐METTL14 markedly inhibited the lactate‐induced concentration of m6A (Figure 4Q). Western blot demonstrated a substantial decline in VSIG4 protein expression after si‐METTL14 treatment (Figure 4R). YTHDC1, an essential m6A reader protein, plays a pivotal role in modulating mRNA stability. Following the knockdown of YTHDC1, we investigated the response of VSIG4 mRNA levels to lactate stimulation. The results showed that lactate markedly stimulated the upregulation of VSIG4 mRNA expression, but si‐METTL14 or si‐YTHDC1 treatment significantly alleviated this upregulation (Figure 4S). Furthermore, we observed that the treatment of si‐METTL14 and si‐YTHDC1 substantially diminished the enhancement of VSIG4 stability induced by lactate (Figure 4T). In conclusion, these findings suggest that CRC cells secrete lactate to promote histone lactylation of macrophages, thereby facilitating the up‐regulation of METTL14 expression and enhancing m6A modification levels. This cascade ultimately contributes to increased VSIG4 expression in macrophages by augmenting the stability of the VSIG4 RNA.

### 3.6 M2 polarization induced by VSIG4 is dependent on fatty acid metabolism

**FIGURE 5:**
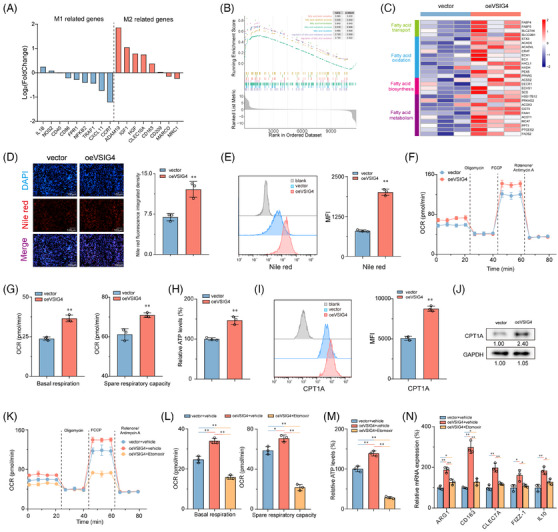
M2 polarization induced by VSIG4 is dependent on fatty acid metabolism. (A) In oeVSIG4 M2‐polarized THP‐1 monocytes and control M2‐polarized THP‐1 monocytes, gene expression analysis was used to detect the expression of M1 and M2 macrophage‐related genes. (B) GSEA analysis of RNA‐seq data showed that fatty acid metabolism‐related pathways were significantly enriched in the oeVSIG4 group. (C) Heatmap view of fatty acid metabolic gene expression in oeVSIG4 M2‐polarized THP‐1 monocytes and control M2‐polarized THP‐1 monocytes. (D) Representative images of Nile red fluorescence staining of M2‐polarized THP‐1 monocytes after VSIG4 overexpression. (E) The expression of Nile red in oeVSIG4 M2‐polarized THP‐1 monocytes and control M2‐polarized THP‐1 monocytes was detected by FCM. (F, G) OCR and SRC of oeVSIG4 M2‐polarized THP‐1 monocytes and control M2‐polarized THP‐1 monocytes were measured by Seahorse XFe 97 analyzer (*n* = 3). (H) ATP production in oeVSIG4 M2‐polarized THP‐1 monocytes and control M2‐polarized THP‐1 monocytes (*n* = 3). (I) The expression of CPT1A in macrophages was detected by FCM. (J) CPT1A protein expression in macrophages was detected by Western blot analysis. (K, L) The effects of the FAO inhibitor (Etomoxir) on OCR and SRC of oeVSIG4 M2‐polarized THP‐1 monocytes and control M2‐polarized THP‐1 monocytes were measured by Seahorse XFe 97 analyzer (*n* = 3). (M) ATP production in oeVSIG4 M2‐polarized THP‐1 monocytes with or without Etomoxir and control M2‐polarized THP‐1 monocytes (*n* = 3). (N) qRT‐PCR was used to detect the effect of Etomoxir on the mRNA levels of M2 macrophage marker genes in oeVSIG4 M2‐polarized THP‐1 monocytes and control M2‐polarized THP‐1 monocytes. *p < .05, **p < .01.

Next, to uncover the molecular mechanism by which VSIG4 promotes macrophage M2 polarization, RNA sequencing (RNA‐seq) was performed on oeVSIG4 and control M2 macrophages, and more than 200 DEGs were identified. Of these, 126 were upregulated, and 129 were downregulated, as depicted in Figure S8A,B. We found that most M2 macrophage‐associated genes were upregulated in the oeVSIG4 group, while M1 macrophage‐associated genes were downregulated (Figure 5A). Additionally, gene set enrichment analysis (GSEA) of the RNA‐seq data revealed a significant enrichment of fatty acid metabolism‐related pathways in the oeVSIG4 group (Figure 5B). Intriguingly, the oeVSIG4 group had higher expression of genes related to the fatty acid metabolic pathway compared with the control group (Figure 5C).

Considering the well‐established impact of fatty acid metabolism on macrophage polarization,^14^ we sought to investigate whether VSIG4‐mediated M2 polarization is achieved via the regulation of fatty acid metabolism. Remarkably, the oeVSIG4 group contained more lipid droplets than the control group (Figure 5D,E). In metabolic assay experiments, oeVSIG4 M2‐polarized THP‐1 monocytes had enhanced oxygen consumption (OCR) and significantly increased spare respiratory capacity (SRC) (Figure 5F,G). Furthermore, oeVSIG4 M2‐polarized THP‐1 monocytes had higher ATP production (Figure 5H). Conversely, VSIG4 knockdown significantly reduced the level of lipid droplet accumulation and metabolism in M2‐polarized THP‐1 monocytes (Figure S8C–G). Mitochondrial carnitine palmitoyl transferase‐1A (CPT1A) is an essential rate‐limiting enzyme for FAO.^37^ We found that VSIG4 overexpression significantly increased CPT1A expression in THP‐1 monocytes compared with the control group (Figure 5I,J). However, siVSIG4 elicited a significant downregulation of CPT1A protein expression (Figure S8H). Intriguingly, treatment with Etomoxir, a CPT1A inhibitor, significantly reversed the accumulation of lipid droplets and increased metabolic levels in THP‐1 monocytes induced by VSIG4 overexpression (Figure 5K,M; Figure S9A). In addition, Etomoxir inhibited the expression of M2 macrophage‐associated genes in oeVSIG4 THP‐1 monocytes (Figure 5N), revealing that inhibition of FAO could alleviate the M2 polarization of macrophages caused by VSIG4 overexpression. Furthermore, to preclude potential off‐target effects associated with Etomoxir, we performed targeted genetic knockdown of Fatty Acid Binding Protein‐4 (FABP4), a pivotal regulator of macrophage lipid metabolism, to confirm these findings. Strikingly, FABP4 knockdown resulted in a marked attenuation of both metabolic flux and the polarization capacity of macrophages towards the M2 phenotype (Figure S9B–F). Taken together, these results suggest that fatty acid metabolism is essential in M2 activation induced by VSIG4 overexpression.

### 3.7 VSIG4 promotes FAO by activating the JAK2/STAT3 pathway to up‐regulate PPAR‐γ

**FIGURE 6:**
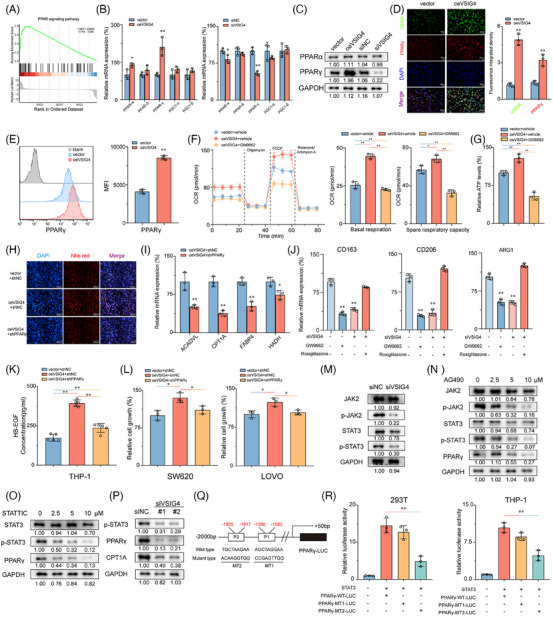
VSIG4 promotes FAO by activating the JAK2/STAT3 pathway to up‐regulate PPAR‐γ. (A) GSEA demonstrating the enrichment of signature genes of PPAR signalling pathway in oeVSIG4 M2‐polarized THP‐1 monocytes. (B) The mRNA levels of PPAR‐α, PPAR‐δ, PPAR‐γ, PGC‐1α, and PGC1‐β in macrophages were detected by qRT‐PCR. (C) Protein expression of PPAR‐α and PPAR‐γ in macrophages was detected by Western blot analysis. (D) The expression of VSIG4 and PPAR‐γ in oeVSIG4 or vector M2‐polarized THP‐1 monocytes was analyzed by Immunofluorescence analysis. Immune complexes were detected with a secondary antibody conjugated with Alexa Fluor 555 (red). DNA is stained with DAPI (blue). Scale, 100 µm. (E) The expression of PPAR‐γ in macrophages was detected by FCM. (F) The OCR and SRC of vector+vector, oeVSIG4+vector and oeVSIG4+GW9772 M2 polarized THP‐1 monocytes were determined by Seahorse XFe 97 analyzer (*n* = 3). (G) ATP production in vector+vector, oeVSIG4+vector and oeVSIG4+GW9772 M2 polarized THP‐1 monocytes (*n* = 3). (H) Representative images of Nile red fluorescence staining of M2‐polarized THP‐1 monocytes treated with or without shPPAR‐γ after VSIG4 overexpression. (I) qRT‐PCR was used to detect mRNA levels of ACADVL, CPT1A, FABP4, HADH in oeVSIG4 M2 polarized THP‐1 mononuclear cells with or without shPPAR‐γ treatment. (J) The effect of PPAR‐γ inhibitor (GW9772, 10 µM) or agonist (Rosiglitazone, 2 µM) on M2 marker expression in M2‐polarized THP‐1 monocytes was analyzed by qRT‐PCR. Data were expressed as mean ± SE and analyzed by one‐way ANOVA with Tukey's multiple comparisons. (K) The content of HB‐EGF in M2 macrophages CM in oeVSIG4 or oeVSIG4+shPPAR‐γ treated groups and control groups was detected by ELISA. (L) CCK8 assay was used to observe the effect of VSIG4 on the viability of CRC cells by regulating the secretion of HB‐EGF in macrophages. (M) Western blot analysis was used to detect the expression of JAK2, p‐JAK2, STATS and p‐STST3 in M2‐polarized THP‐1 monocytes treated with control or siVSIG4. (N–O) M2‐polarized THP‐1 macrophages (IL‐4/IL‐13‐stimulated for 72 h) were treated with the JAK2 inhibitor AG490 or STAT3 inhibitor STATTIC for 24 h. Western blotting was performed to assess phosphorylation of JAK2 and STAT3. GAPDH served as the loading control. (P) Western blot analysis was used to detect the protein expression of p‐STST3, PPAR‐γ, and CPT1A in M2‐polarized THP‐1 monocytes treated with control or siVSIG4. (Q) Schematic diagram of PPARγ promoter mutation strategy (−2000 to 50 bp). (R) Luciferase reports showed that STAT3 binds to PPARγ promoter site 2 and induces its expression. *p < .05, **p < .01.

The PPAR pathway is widely recognized as a crucial signalling pathway involved in regulating fatty acid metabolism.^38^ GSEA analysis showed that the PPAR pathway activation was significantly associated with elevated VSIG4 levels in CRC cohorts (Figure 6A). PPAR‐α and PPAR‐γ expressions were significantly increased in oeVSIG4 M2‐polarized THP‐1 monocytes compared with controls, while PPAR‐δ, PGC‐1α, and PGC1‐β expressions were virtually unchanged (Figure 6B). Consistently, the expression of PPAR‐α and PPAR‐γ in M2‐polarized THP‐1 monocytes treated with siVSIG4 decreased significantly, but there was no effect on the expression of PPAR‐δ, PGC‐1α, and PGC1‐β (Figure 6B). However, Western blot analysis showed that VSIG4 levels had an impact on PPAR‐γ protein expression while having a negligible effect on PPAR‐α expression (Figure 6C). Given the significant changes in PPAR‐γ expression, we selected PPAR‐γ for further investigation. Immunofluorescence analysis revealed that VSIG4 overexpression significantly increased the PPAR‐γ expression (Figure 6D), while flow analysis further confirmed the induction effect of VSIG4 on PPAR‐γ expression (Figure 6E). Treatment with GW9662(PPAR‐γ inhibitor) significantly reduced OCR and SCR in M2‐polarized THP‐1 monocytes, accompanied by a decrease in ATP production (Figure 6F,G), suggesting that VSIG4 regulates FAO by modulating PPAR‐γ expression. Consistent with this, shRNA‐mediated knockdown of PPAR‐γ significantly reduced the lipid droplet content in M2‐polarized THP‐1 monocytes (Figure 6H). Knockdown of PPAR‐γ expression resulted in reduced expression of genes involved in the FAO pathway, including CPT1A, HADH, ACADVL, and FABP4 (Figure 6I). In addition, Etomoxir treatment also significantly down‐regulated the expression of these genes (Figure S9G). Subsequently, we investigated the role of PPAR‐γ in M2 polarization of THP‐1 monocytes. GW9662 and siVSIG4 significantly inhibited M2 polarization in THP‐1 monocytes, as manifested by the downregulation of CD163, CD206, and ARG1 expression (Figure 6J). Conversely, Rosiglitazone (PPAR‐γ agonist) reversed the downregulation of M2 marker gene expression (Figure 6J). Notably, knockdown PPAR‐γ also significantly alleviated the increase in HG‐EGF secretion induced by oe‐VSIG4 and the proliferation of CRC cells enhanced by oe‐VSIG4 M2 macrophage CM (Figure 6K,L).

It is well known that the JAK2/STAT3 pathway plays a pivotal role in regulating macrophage M2 polarization.^39^ To investigate whether the JAK2/STAT3 pathway mediates the regulation of macrophages M2 polarization by VSIG4, we employed IL‐13/IL‐4 to stimulate PMA‐activated THP‐1 monocytes that differentially express VSIG4 to promote M2 polarization in M0 macrophages. Our results show that knockdown VSIG4 inhibits JAK2 and STAT3 phosphorylation in macrophages, inducing a modest reduction in STAT3 protein expression while leaving JAK2 protein levels unaltered (Figure 6M). Treatment of M2‐polarized THP‐1 monocytes with either a JAK2 inhibitor (AG490) or STAT3 inhibitor (STATTIC) led to significant suppression of PPAR‐γ expression (Figure 6N,O). Furthermore, knockdown of VSIG4 resulted in decreased PPAR‐γ and CPT1A expression (Figure 6P). To determine whether STAT3 directly stimulates PPAR‐γ expression, we co‐transfected the PPAR‐γ promoter constructs (PPAR‐γ‐WT‐Luc, PPAR‐γ‐MT1‐Luc, PPAR‐γ‐MT2‐Luc) along with the STAT3 expression plasmid into 293T and THP‐1 monocytes (Figure 6Q). The luciferase reporter assay demonstrated that transfection with PPAR‐γ‐WT‐Luc markedly augmented luciferase promoter activity, whereas transfection with PPAR‐γ‐MT2‐Luc, but not PPAR‐γ‐MT1‐Luc, significantly diminished luciferase promoter activity (Figure 6R). These findings robustly suggest that STAT3 directly interacts with the PPAR‐γ promoter, thereby inducing PPAR‐γ transcription in these cell lines. These results suggest that VSIG4 up‐regulates PPAR‐γ expression by activating the JAK2/STAT3 pathway.

### 3.8 Targeted VSIG4 treatment enhances the efficacy of PD‐1 blockade

**FIGURE 7:**
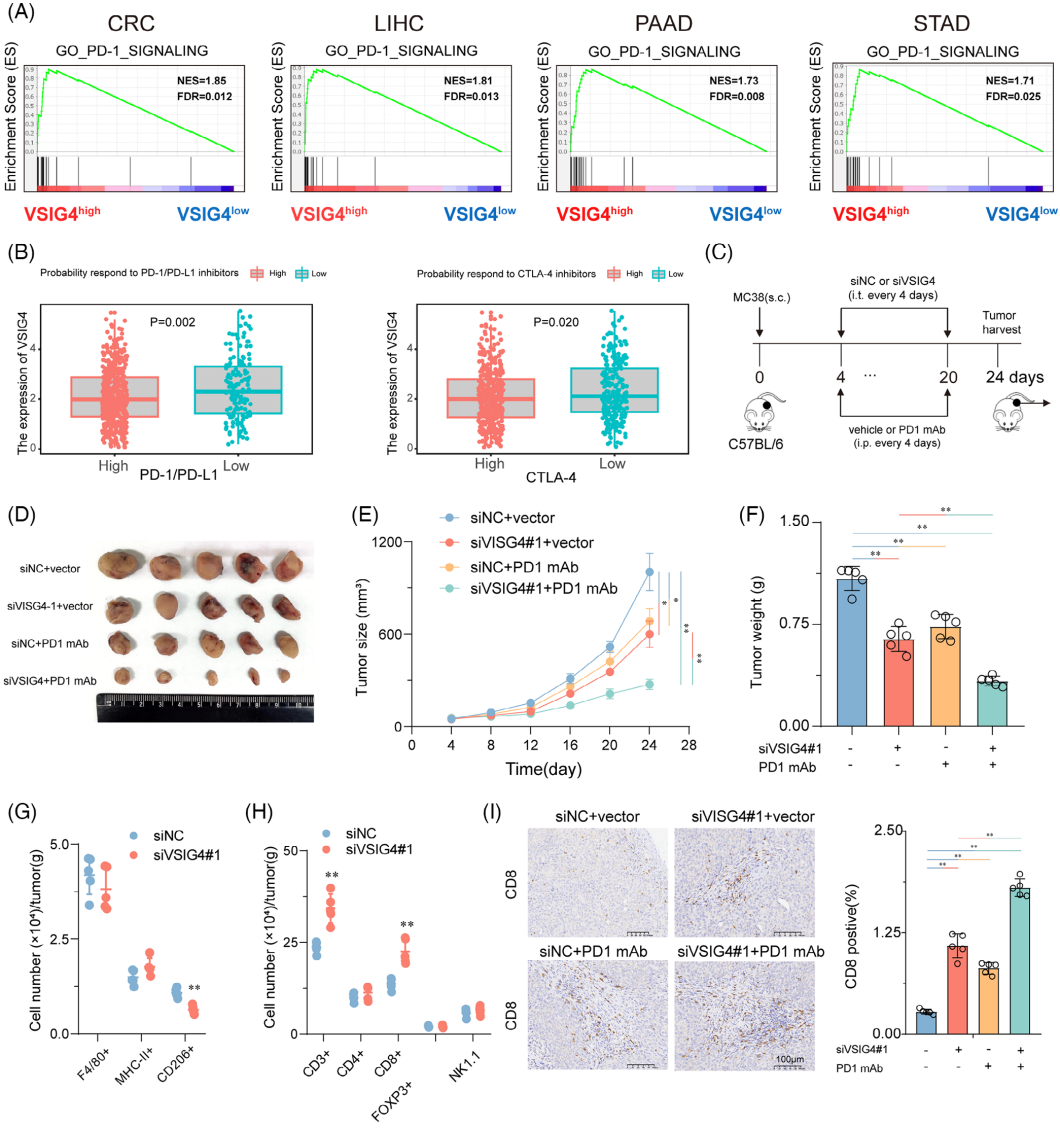
Targeted VSIG4 treatment enhances the efficacy of PD‐1 blockade. (A) GSEA demonstrates the enrichment of signature genes of the PD‐1 signalling pathway in tumours with high VSIG4 expression. (B) The association between VSIG4 expression levels and the probability of response to immunotherapy, including anti‐PD‐1 /PD‐L1 therapy and anti‐CTLA‐4 therapy. (C–F) MC38 cells (5×105) were subcutrally transplanted (s.c.) C57BL/7 mice and treated with isotype‐controlled, in vivo optimized small interfering RNA (siVSIG4), anti‐PD‐1 mAb, or siVSIG4 in combination with anti‐PD‐1 mAb. (C) Schematic diagram of the treatment plan. (D) Representative images of MC38 tumours. (E) Tumour size. (F) Tumour weight. (G) Live CD45^+^ cells were subdivided into macrophages (F4/80^+^), T cells (CD3^+^), and NK cells (NK1.1^+^). M1 (MHC‐II^+^) and M2 (CD206^+^) macrophage subsets were further defined within the F4/80^+^ population. CD8^+^ T cells, CD4^+^ T cells, and Tregs (CD25^+^ Foxp3^+^) were identified within the CD3^+^ population. (I) Representative images of CD8 immunohistochemical staining of subcutaneous tumour sections in each group, *n* = 5. Scale, 100 µm. *p < .05, **p < .01.

Given that VSIG4 inhibits T cell function and proliferation, we hypothesized that obstructing VSIG4 could potentially enhance the efficacy of anti‐PD‐1/PD‐L1 treatment. To this end, VSIG4 co‐expression patterns with immune checkpoints (PD‐L1, PD‐1, CTLA‐4, FOXP3, LAG3, TIGIT, TIM3) were analyzed. The results showed that VSIG4 in CRC was positively correlated with multiple immune checkpoints (Figure S10A). Moreover, GSEA revealed significant enrichment of PD‐1 signalling in VSIG4^high^ tumours, emphasizing the positive correlation between VSIG4 and PD‐1 pathway (Figure 7A). Notably, we also investigated the correlation between VSIG4 expression levels and immune predictive scores in CRC patients, aiming to predict their response to immune checkpoint inhibitors (ICIs). Our findings revealed that CRC patients with increased VSIG4 expression exhibited a diminished response to immunotherapy, such as anti‐PD‐1/PD‐L1 (*p* = .002) and anti‐CTLA4 (*p* = .020) therapy (Figure 7B). Furthermore, a thorough exploration of the connection between patient response to antiPD‐1/PD‐L1 treatment and VSIG4 expression levels was undertaken across various other cancers (LUAD, PAAD, KIRC, and STAD). The results illustrated that in these malignancies, individuals with low VSIG4 expression profiled themselves as better candidates for immunotherapy (Figure S10B).

Subsequently, we validated the above analysis through animal experiments by utilizing vivo‐optimized siRNA targeting the VSIG4 gene combined with anti‐PD‐1 monoclonal antibody (anti‐PD‐1 mAb) to treat MC38 tumour‐bearing mice (Figure 7C). We found that treatment with anti‐PD‐1 mAb or siVSIG4 alone could inhibit tumour growth. However, the combined treatment of both exhibited even greater inhibition of tumour growth in the mice (Figure 7D–F). In addition, combination therapy significantly improved the TIME. Inhibition of VSIG4 did not alter the total number of macrophages and the proportion of M1 macrophages in the TIME, but it significantly reduced the proportion of M2 macrophages (Figure 7G). Moreover, knockdown VSIG4 significantly increased the infiltration of T cells (CD3^+^) in the TIME, particularly CD8^+^ T cells (CD8^+^), while having no effect on the infiltration of Tregs (CD25^+^FOXP3^+^), CD4^+^ T cells (CD4^+^), and NK cells (NK1.1^+^) (Figure 7H). IHC analysis of CD8^+^ cell infiltration revealed that monotherapy with either anti‐PD‐1 monoclonal antibodies or siVSIG4 induced a marked elevation in intratumoral CD8^+^ T cell density. Notably, the combinatorial regimen demonstrated synergistic effects, producing superior T‐cell accumulation compared with individual treatments (Figure 7I). These results demonstrate VSIG4 inhibition as a potential therapeutic approach to amplify immunotherapy responses in CRC.

### 3.9 VSIG4 and CD8A are valuable prognostic indicators in patients with CRC

**FIGURE 8:**
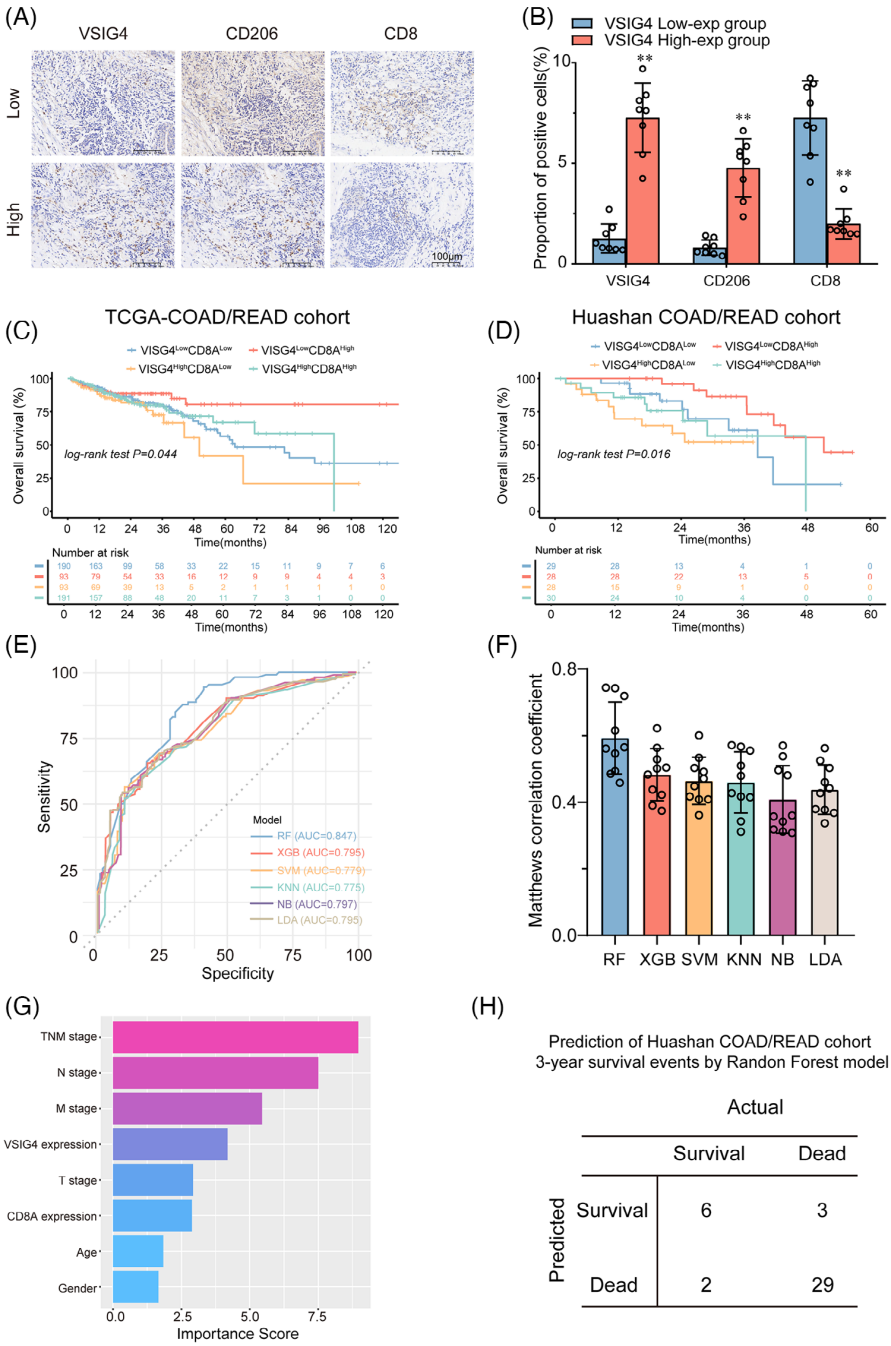
VSIG4 and CD8A are valuable prognostic indicators in patients with CRC. (A) Immunohistochemical staining was performed on 8 clinical specimens randomly selected from two groups of colorectal cancer patients with different VSIG4 expression levels, and representative images of VSIG4, CD206 and CD8 expression were obtained. (High: VSIG4 high expression group, low: VSIG4 high expression group). Scale, 100 µm. (B) Quantitative histogram of immunohistochemical staining results. In the COAD/READ cohort (C) of the TCGA database and the COAD/READ cohort (D) of Huashan Hospital, the Kaplan–Meier method was used to plot the OS curve according to the VSIG4 and CD8A expression. Patients with high expression of VSIG4 and low expression of CD8A had significantly lower OS (*p* = .044 (C); *p* = .017 (D)). (E) ROC curve analysis of five machine learning algorithms for predicting the 3‐year survival probability of COAD/READ patients in the TCGA database. (F) Matthews correlation coefficients for five machine learning algorithms. (G) Importance ranking diagram of clinical parameters based on the XGB model. (H) The established random forest model was used to predict the 3‐year survival outcomes of forty patients in the Huashan COAD cohort. *p < .05, **p < .01.

To further explore the relationship between VSIG4 levels and macrophage polarization and CD8^+^ T infiltration in CRC patients, IHC staining was performed on human CRC samples from Huashan Hospital. The samples were stratified by VSIG4 expression into high/low groups. VSIG4 expression exhibited a positive correlation with M2 macrophages but a negative association with CD8^+^ T cell infiltration across CRC specimens (Figure 8A,B). Additionally, we discovered that high VSIG4 expression levels and reduced CD8A expression were associated with poor overall survival (OS) in patients (Figure 8C,D), suggesting that CD8A and VSIG4 may serve as valuable prognostic indicators for CRC patients. To further explore the prognostic impact of VSIG4 and CD8A expression in patients with CRC, 205 patients from the TCGA‐Coad/Read cohort were analyzed using five machine learning algorithms for 3‐year survival prediction. With the highest AUC (.847) and Matthews correlation coefficient (.55) in 3‐year survival prediction, Random Forest demonstrated superior performance, establishing it as the optimal predictive model (Figure 8E,F). Then we extracted the feature importance values of various clinical parameters via the Random Forest model, subsequently ranking them according to their scores to elucidate the significance of each clinical parameter. The Random Forest model ranked CD8A and VSIG4 gene expressions fourth and sixth, respectively, in importance scoring (Figure 8G). To determine the model efficacy, we screened patients from the Huashan‐COAD cohort who survived for more than three years and those who died within three years (40 cases). Subsequently, we employed the pre‐existing random forest model to predict the 3‐year survival outcomes for these 40 patients. By comparing these predictions with the clinical follow‐up data, we observed that our constructed model achieved a validation cohort prediction accuracy of 87.5% (Figure 8H). Furthermore, we have developed an internet‐based computational tool utilizing the most effective models (https://huashanpharmacy.shinyapps.io/VSIG4_CRC/), enabling healthcare professionals to estimate the likelihood of a patient's five‐year survival by conveniently entering the patient's pathologic variables and the expression levels of VSIG4 and CD8A.

## 4 DISCUSSION

VSIG4 belongs to the B7 family, one of the most critical inflammatory protein families, and is only expressed by macrophages.^23^ VSIG4 expression has been detected in the tissues of patients afflicted by rheumatoid arthritis and atherosclerosis (AS), which led to speculation that it could potentially be involved in the pathogenesis of inflammatory diseases.^40^ Recent investigations have revealed that the expression of VSIG4 is upregulated in a diverse array of malignancies, indicating its promising potential as a therapeutic target in oncology.^25^, ^26^, ^27^, ^28^ While the immunosuppressive implications of VSIG4 in TIME have been unveiled across various malignancies,^23^ the known mechanisms of VSIG4 in immunosuppression to date are direct inhibition of IL‐2 production, effect of CD8^+^ T cell proliferation, and induction of Tregs.^41^ Here, we found that VSIG4, as a macrophage‐specific protein, can indirectly inhibit T cell function by regulating the polarization of macrophages. In murine experiments, targeted knockdown of VSIG4 resulted in inhibited tumour growth, accompanied by a reduction in the proportion of M2‐TAMs and exhausted T cells, alongside enhanced activity of CD8^+^ T cells. However, we have not yet systematically assessed the impact of siVSIG4 on immune cell populations within the peripheral circulation. Consequently, a critical question remains unresolved: whether the enhanced intratumoral CD8^+^ T cell infiltration observed following siVSIG4 treatment arises from amplified recruitment signalling within the tumour microenvironment or, alternatively, via an expansion of circulating CD8^+^ T cell populations in peripheral blood. Subsequent investigations will rigorously evaluate the systemic immunological consequences of siVSIG4, specifically its influence on peripheral CD8^+^ T cell abundance, to elucidate the mechanistic basis of its antitumor efficacy. Additionally, the observed enhancement of CD8^+^ T cell activity was reversed following treatment with clodronate liposomes, demonstrating that the inhibition of T cells by VSIG4 is macrophage‐dependent. Notably, clodronate liposomes not only target to deplete macrophages but also inadvertently affect certain monocytes and dendritic cells, potentially leading to off‐target effects, which is the limitation of this experiment. Moreover, the significant alleviation of tumour growth inhibition after the neutralization of CD8^+^ T cells with CD8^+^ T antibodies suggests that the protumour function of VSIG4 is partially achieved by creating inhibitory TIME.

TAMs constitute a critical component of the tumour‐infiltrating immune cells, with the majority displaying characteristics emblematic of M2 macrophages.^9^ Diverse therapeutic strategies have emerged to target TAMs, falling into two main categories.^42^ One is to reduce or eliminate TAMs content in TIME by reducing TAMs recruitment or promoting TAMs apoptosis, such as inhibiting TAMs recruitment by blocking the CCL2‐CCR2 axis or inducing TAMs apoptosis by blocking the CSF1‐CSF1R axis.^43^, ^44^ However, macrophages, as one of the antigen‐presenting cells in solid tumours, play a crucial role in processing and presenting the antigens on tumour cells to T cells, and eliminating TAMs would result in the alleviation of this vital function.^45^ Therefore, more attention has been paid to the second strategy, that is, to reprogram the immunosuppressive TAMs into immunostimulatory TAMs. Specifically, this approach focuses on re‐polarizing M2 macrophages into M1 macrophages or reducing the proportion of M2 macrophages to inhibit tumour growth and metastasis.^46^ For example, by MS4A4A blockade treatment, M2 TAMs infiltration is reduced and effector CD8^+^ T cells infiltration is increased, thereby reshaping the TIME.^47^ Furthermore, it has been established that M2 macrophages facilitate the proliferation of tumour cells while concurrently inhibiting their apoptosis through the secretion of HB‐EGF.^48^ For instance, Molly J Carroll et al have shown that M2 macrophages can stimulate the proliferation of ovarian cancer cells via the HB‐EGF/MMP9 intercellular signalling pathway.^49^ Here, we found that inhibition of VSIG4 in vitro alleviates the polarization of macrophages towards the M2 subtype, while overexpression of VSIG4 promotes this process. In addition, CM from siVSIG4‐pretreated M2 macrophages significantly alleviated the proliferation, migration, and invasion of LOVO and SW620 tumour cells and inhibited their apoptosis. In vivo, inhibition of VSIG4 also reduced the proportion of M2 TAMs and inhibited tumour growth.

While ICIs treatment has yielded enduring and favourable outcomes in tumours such as melanoma and lung cancer, their impact is confined to individuals with specific tumour types. In PDAC, for example, ICIs have exhibited limited efficacy, primarily relegated to clinical trials or serving as a salvage treatment for advanced‐stage patients.^50^ The underlying mechanisms contributing to the low reactivity of ICIS include low mutation load, effector T cell exhaustion or insufficient infiltration, and inhibitory immune cell infiltration.^51^ Presently, ICIs treatment demonstrates efficacy solely in patients with metastatic CRC exhibiting mismatch repair defects and high microsatellite instability (dMMR‐MSI‐H), while displaying poor therapeutic efficacy in patients with microsatellite stable CRC.^52^ Consequently, there exists an urgent need to develop novel therapeutic strategies that could broaden the spectrum of CRC patients poised to benefit from immunotherapy. A prerequisite for effective immunotherapy is the infiltration of T cells in the TIME.^53^ However, the immune checkpoints discovered so far (PD‐1, CTLA‐4, T cell immunoglobulin and mucin‐domain‐containing protein 3 (TIM3), lymphocyte activation gene 3 (LAG‐3), and T‐cell immunoglobulin receptor with IG and ITIM domains (TIGIT)) are mainly concentrated on T cells.^54^, ^55^, ^56^, ^57^ Targeting immune checkpoints on T cells alone is not sufficient to achieve an effective therapeutic outcome, so a combination strategy is needed to achieve significant antitumour efficacy. In light of the critical role played by macrophages in inhibiting the function of CD8^+^ T cells, the current focus predominantly revolves around strategies that target TAMs therapy combined with ICI therapy.^58^ Our findings reveal that VSIG4 orchestrates FAO via the JAK2/STAT3/PPARγ axis, thereby establishing a metabolically immunosuppressive TAMs phenotype. This links macrophage metabolism to CD8^+^ T cell dysfunction and immune escape. Targeting VSIG4 not only disrupts FAO‐dependent M2 polarization but also synergistically enhances tumour susceptibility to PD‐1 blockade. Promising therapeutic strategies may involve the combinatorial administration of VSIG4 inhibitors or FAO inhibitors with ICIs to amplify synergistic efficacy. Furthermore, metabolomic profiling of VSIG4‐high tumours could unveil predictive biomarkers, thereby facilitating personalized therapeutic regimens for stratified patient cohorts through precision medicine approaches.

## 5 CONCLUSION

In conclusion, our findings elucidate the key role of VSIG4^+^TAMs in modulating immune escape in CRC, suggesting that targeting macrophage VSIG4 could significantly improve the efficacy of anti‐PD‐1 therapy. Mechanistically, CRC cells release lactate to upregulate the expression of VSIG4 in macrophages through epigenetic modification, and VSIG4 promotes PPAR‐γ expression and FAO of macrophages by regulating the JAK2/STAT3 signalling pathway, thus promoting macrophages towards M2 polarization . Collectively, our study identifies the clinical value of VSIG4 blockade in reshaping the immune microenvironment of immunosuppressive tumours and provides a novel perspective for understanding the role of TAMs in modulating anti‐tumour immunity.

## AUTHOR CONTRIBUTIONS

Jiafeng Liu, Tianxiao Wang, WenXin Zhang, and Qunyi Li designed the research. Tianxiao Wang, WenXin Zhang, Xiang Mao, Li Chen, and Xinhai Wang collected samples and clinical data. Jiafeng Liu, Tianxiao Wang, Yuxin Huang, and Zimei Wu performed the bioinformatic analysis. Jiafeng Liu, Tianxiao Wang, WenXin Zhang, Huanying Shi, Li Chen, Qunyi Li, and Huijie Qi analyzed the data. Qunyi Li, Mingkang Zhong, and Xiaojin Shi supervised the study. Jiafeng Liu and Tianxiao Wang wrote the manuscript. Qunyi Li, Tianxiao Wang, WenXin Zhang, Huanying Shi, Lu Chen, and Zimei Wu reviewed the manuscript. Tianxiao Wang, Li Chen, Qunyi Li, WenXin Zhang, and Huanying Shi acquired the funding. All authors agreed to be accountable for all aspects of the work ensuring integrity and accuracy.

## CONFLICT OF INTEREST STATEMENT

The authors declare no conflict of interest.

## CONSENT FOR PUBLICATION

Not applicable.

## ETHICS STATEMENT

The collection of patients’ samples was approved by the Ethics Committee of Huashan Hospital (approval number: KY2021‐472). All animal procedures were performed according to the approved protocol by the Fudan University Institutional Animal Care and Use Committee (approval number: 2023‐HSYY‐118JZS).

## Supporting information



Supporting Information

## Data Availability

All data generated or analyzed during this study are included in this published article (and its supplementary information files).
