# Supplementary material for: VSIG4 Promotes Tumour‐Associated Macrophage M2 Polarization and Immune Escape in Colorectal Cancer via Fatty Acid Oxidation Pathway
Source: Clin Transl Med. 2025 May 22;15(5):e70340. doi: 10.1002/ctm2.70340 (PMC12098961; doi:10.1002/ctm2.70340)
Supplement: Supplementary file 1 — Supporting Information [file CTM2-15-e70340-s001.docx]

**Supplemental material**

**Materials and Methods**

Reagents

Clodronate Liposomes (40337ES10) was purchased from Yeasen biotechnology (Shanghai, China), Etomoxir (HY-17538), oligomycin (HY-N6782), carbonyl cyanide 4-(trifluoromethoxy) phenylhydrazone (HY-100410), Rotenone (HY-B0389) were purchased from Med Chem Express (USA). Antimycin A (1397-94-0) was purchased from Santa Cruz Biotechnology. These reagents were stored at –20 °C.

Cell viability assay

The Cell Counting Kit-8 (Proteintech Group, Inc, USA) was employed to perform a cell viability assay. Specifically, CRC cells were seeded into 96-well plates at a concentration of 6 × 10^3^ cells/well and incubated under standard culture conditions for 24 h. A 10 µL of CCK-8 solution was added to each well and incubated for 1 h at 37 °C per the protocols. A microplate reader (Biotek, Winooski, VT, USA) was utilized to measure the optical density (OD) values at 450 nm. Each experiment was conducted independently three times, with five replicates each.

Macrophage polarization and preparation of conditioned media

THP-1 monocytes differentiated into M0 macrophages by incubating in 100ng/ml PMA for 24h. To obtain M1 subtype macrophages, THP-1 monocytes were treated with 100ng/ml PMA for 24h and then cultured with 100ng/ml lipopolysaccharide (LPS) and 20ng/ml IFN-γ for 48h. To obtain M2 subtype macrophages, THP-1 monocytes were treated with 100ng/ml PMA for 24h and then cultured with 20ng/ ml IL-13 and 20ng/ ml IL-4 for 48h.

Macrophages with different polarization states were washed with PBS and incubated in serum-free medium for 24h. The medium was collected and then centrifuged at 10,000 rpm for 5 min, filtered to remove cell debris, and the culture supernatant was recovered and stored at -80℃ for later use.

Transfections

Lentiviral vectors of VSIG4 overexpression and negative control were obtained from GeneChem Co (Shanghai, China), and transfected into THP-1 cells. Lentiviral transfection procedures were performed according to provided protocols. Human and mouse VSIG4 siRNA was synthesized by Hanbio Technology (Shanghai, China).

Human:

siNC: Antisense: 5'-AUCGAUGAUAUACUAUACGUGdTdT-3'

Sense: 5'-CAUAGUAUAUCAGUCGAUAAC-3'

siVSIG4#1: Antisense: 5'-UGCUUAUACCAAAUAUAACUGdTdT-3'

Sense: 5'-GUUAUAUUUGGUAUAAGCAAC-3'

siVSIG4#2: Antisense: 5'-AACAUUAGACUUAUAGUGGAGdTdT-3'

Sense: 5'-CCACUAUAAGUCUAAUGUUCU-3'

Mouse:

siNC: Antisense: 5'-AUCGAUGAUAUACUAUACGUGdTdT-3'

Sense: 5'-CAUAGUAUAUCAGUCGAUAAC-3'

siVSIG4#1: Antisense: 5'-AAGAUUAUGGCAAAGAUUGGCdTdT-3'

Sense: 5'-CAAUCUUUGCCAUAAUCUUCA-3'

siVSIG4#2: Antisense: 5'-AUAAGUAACAGGUAAUUACGCdTdT-3'

Sense: 5'-GUAAUUACCUGUUACUUAUUU-3'

siRNA transfection was performed. Briefly, THP-1 cells (6 × 10^5^) were cultured in six-well plates, 50 nM siRNA was mixed with LipofectAMINE 2000 Transfection Reagent (Life Technologies, Carlsbad, CA) and added to the wells of six-well plates and incubated for 24 hours. Cells were incubated for 48 or 72 hours after transfection before testing for transgene expression or performing downstream experiments.

M6A dot blot assay

The m6A RNA Methylation Quantification kit (Epigentek) was used to determine the levels of m6A in total RNA. The extracted RNA samples were quantified, 200 ng RNA was used for each detection hole, the RNA sample points were placed on the Amersham Hybond-N+ membranes, and the membranes were left to dry at room temperature. Incubation at room temperature for 1 hour with a sealer solution (5% BSA) and overnight incubation at 4°C with m6A antibodies (1:1000, Synaptic Systems, catalog number 202003). Wash the membranes with PBS three times for 7 minutes each time. Add the secondary antibody and incubate at room temperature for 1 hour. M6A levels were measured using an ECL chemiluminescence kit (Tanon). The NC membranes were stained with methylene blue and then washed with ddH2O until clear spots appeared.

Western blot analysis and ELSIA

Western blotting was performed as described previously. The primary antibodies used were GAPDH (#2118), CPT1A (#12252), JAK2 (#3230), p-JAK2 (#3776), PPAR-γ (#2435), STAT3 (#12640), p-STAT3 (#9145) (Cell Signaling Technology), and VSIG4 (PA4-42027) (Invitrogen). These primary antibodies were followed by appropriate secondary antibodies conjugated to horseradish peroxidase. Antibody-protein complexes were detected using enhanced chemiluminescence (ECL) immunoblotting detection reagent. The signals were analyzed using a LAS-3000 image analyzer and MultiGauge software (Fuji Film). Chemokine concentrations of TNF-β and IL-10 were measured using ELISA kits (Invitrogen) based on the manufacturer’s instructions.

Measurement of lactate levels

PMA-activated THP-1 monocytes or control cells supernatant were collected from the co-culture system by centrifugation at 1500 rpm for 10min, and lactate levels were measured using colorimetry according to the manufacturer's instructions (Sangon Biotech, catalog number D799851).

ChIP-qPCR

The chromatin immunoprecipitation (ChIP) determination was performed using the Simple ChIP Plus Sonication Chromatin IP Kit (Cell Signalling Technology) according to the manufacturer's instructions. PMA-activated THP-1 monocytes were incubated with 1% formaldehyde at 37°C for 10 min. Glycine was added (final concentration 125 mM) to terminate the reaction, cells were collected after incubation for 5 min.The cells were re-suspended using lysis buffers and the chromatin was clipped to a suitable fragment size (200-1000 bp) by ultrasonic treatment. The cleavage products were diluted in a buffer containing a protease inhibitor. Specific antibodies are added and incubated overnight at 4°C to form antibody-protein-DNA complexes. Protein A/G agarose beads were added and incubated at 4°C for 2 hours to precipitate the complex. The precipitate was added to the lysis buffer, NaCl (0.3 M) was added and incubated at 65°C for 2 hours to reverse the crosslinking. DNA was extracted using a DNA purification kit. The product was quantified by qPCR.

MeRIP-qPCR

MeRIP was performed using the riboMeRIP m6A Transcriptome Profiling Kit (RiboBio, catalog number C11051-1). Total RNA was extracted with RNA extraction kit, and lysed RNA with lysis buffer. M6A antibodies were then mixed with RNA samples, incubated on ice for 1 hour, added magnetic beads, and continued to incubate for 2 hours. The supernatant was then digested with a proteinase K buffer. A mixture of phenol-chloroform-isoamyl alcohol was used to extract RNA conjugated by antibody microspheres. The purified RNA was reversely transcribed into cDNA using reverse transcription kit according to the kit instructions, and real-time quantitative PCR was performed.

Nile red staining

For the quantification of lipid droplets in cells, Nile red staining was employed. M2-polarized THP-1 monocytes were seeded into 6-well plates, each containing a pre-placed sterile glass coverslip. Once cell confluence reached between 60% and 80%, the cells were fixed in 2 ml of 2% formaldehyde in PBS for 20 minutes. Subsequently, the cells were incubated with 1 ml of Nile red staining solution (Beyotime, China) for 20 minutes, followed by staining with DAPI (Beyotime, China) for 20 minutes at room temperature. The coverslips were then mounted onto glass slides using 30 μl of antifade mounting medium. All images were captured and analyzed using a Leica DM6B microscope. Quantification of the Nile red content was conducted via flow cytometry.

Measurement of OCR and ATP Content

Oxygen consumption rate was quantified using a Seahorse Bioscience XF24 Extracellular Flux Analyzer (Seahorse Bioscience). M2-polarized THP-1 monocytes cells (1x10^4^) was seeded into specialized V7 Seahorse tissue culture plates and incubated overnight. Cells were then incubated in a non-CO2 incubator for 1 hour. Following the measurement of basal oxygen consumption rate (OCR), Oligomycin, FCCP, and Antimycin/Rotenone were sequentially injected into the cell chamber as per Seahorse standard protocol. M2-polarized THP-1 monocytes cells (1×10^6^) were seeded into a 6-well plate and cultured for 24 h until cells adhered. ATP levels were assessed per the ATP Assay kit instructions (Sangon Biotech, Shanghai, China). The obtained results were adjusted based on cell numbers to ensure accuracy. All measurements were performed in three wells per condition per experiment and repeated at least three times.

Quantitative real‑time polymerase chain reaction

Total RNA was isolated using the Trizol reagent and Ultrapure RNA kit (CW Biotech, China) according to the manufacturer's instructions. A 2 mg of total mRNA was reverse-transcribed into cDNA using the Superscript™ reverse transcription system (Takara, Kyoto, Japan). Quantitative real-time reverse transcription polymerase chain reaction (RT-PCR) reactions were performed on an ABI 7500 Real-Time PCR system (Applied Biosystems, Foster City, CA, USA) using SYBR Green PCR master mix reagents (Takara, Kyoto, Japan). Relative quantification of the target gene was calculated after being normalized to GAPDH gene expression using the 2^-△△Ct^ method. The primer sequences were selected based on the RTPrimerDB database (<http://medgen.ugent.be/rtprimerdb/>).

RNA-Seq and data analysis

Total RNA was extracted from THP-1 cells with VSIG4 overexpression and control cells using Trizol reagent (Invitrogen, Carlsbad) following the protocol. The quality and quantity of RNA were assessed using a NanoDrop ND-1000 spectrophotometer. The cDNA library was prepared using the MGI Stranded RNA-Seq Library Preparation Kit per the manufacturer's instructions. RNA sequencing was performed on an MGI MGISEQ-2000 system following protocols. To gain further insight into the biological pathways involved in lipid metabolism related to VSIG4, gene set enrichment analysis (GSEA) was conducted.

Tumor immune phenotyping and flow cytometry analysis

Tumors were extracted and mechanically dissociated into fragments within 2 hours. Subsequently, the tumor tissue was disrupted to prepare single cells using a tumor isolation kit (Miltenyi Biotec) according to the manufacturer’s instructions. The cell suspension was filtered through a 70 μm cell strainer to remove red blood cells. Tumor-infiltrating leukocytes were isolated via gradient centrifugation employing a 40%/80% Percoll (GE Healthcare) solution. Thereafter, the collected cells were incubated with an Fc block (anti-mouse CD16/32, BioLegend) on ice for 30 minutes. The samples were initially stained for surface markers of lymphoid immune populations prior to intracellular staining. For intracellular staining, the True-Nuclear Transcription Factor Buffer Set 424,401 (BioLegend) was utilized according to the manufacturer’s guidelines. The following antibodies and stain kit were procured from BioLegend: APC-Cy7 anti-mouse CD45 (cat#103116), BV510 anti-mouse CD3 (cat#100234), PerCP-Cy5.5 anti-mouse CD4 (cat#100434), FITC anti-mouse CD8a (cat#100706), PE anti-mouse IFN-γ (cat#505808), PE-Cy7 anti-mouse PD-1 (cat#135215), APC anti-mouse TIM3 (cat#119705), PE-Cy7 anti-mouse F4/80 (cat#123122), PE anti-mouse CD206 (cat#1411706), PerCP-Cy5.5 anti-mouse NK1.1 (cat#108728), BV421 anti-mouse CD11b (cat#101235). FITC anti-mouse MHC-II (cat#11-5321-82) was purchased from eBioscience (Thermo Fisher Scientific, Waltham, MA).

Dual-luciferase assays

To assess the activity of the −2000 to +50 regions of the PPARγ promoter, dual-luciferase assays were conducted. Individual groups of cells were co-transfected with plasmids for a single fragment in the region to control luciferase expression and the Renilla luciferase reporter for 24 h. Following the transfection, the levels of firefly and Renilla luciferase activities were assessed using the Dual-Luciferase Reporter Assay System (Promega).

Histopathology and immunohistochemistry analyses

To evaluate VSIG4, CD206 and CD8A expression in patient tissue sections, the sections were subjected to antigen retrieval by heating in a microwave oven at 100 °C for 15 min using citrate buffer (pH 6.0) after deparaffinization and rehydration. The sections were incubated with VSIG4 antibody (1:200, PA5-52018, Invitrogen), CD206 antibody (1:1000, PA5-147678, Invitrogen) and CD8A antibody (1:200, MA5-14548, Invitrogen) overnight, respectively, at 4 °C. Subsequently, a secondary antibody (goat anti-rabbit conjugated to horseradish peroxidase, 1:200; #ab97051, Abcam) was applied for 1 h at room temperature. The sections were stained using 3,3′-diaminobenzidine tetrahydrochloride (Long Island, Shanghai, China), and VSIG4, CD206 and CD8A expression was assessed based on staining intensity and extent.

Machine learning feature assessment

Selected features were assessed using six machine-learning algorithms: Random Forests (RF), Support Vector Machines (SVM), eXtreme Gradient Boosting (XGB), K Nearest Neighbors (KNN), Naive Bayes (NB) and Linear Discriminant Analysis (LDA). The prediction was performed using mlr3 package (version 0.16.1) from R language. To evaluate the performance of selected features in TCGA-COAD cohort (n = 205), 10-fold cross validation generated six models and the area under the curve (AUC) for the receiver operating characteristic (ROC) was obtained.

WGCNA

Weighted gene co-expression network analysis (WGCNA) is a systembiology approach for clustering highly correlated genes into a module and calculating the magnitude of the correlation between different modules and selected clinical phenotypes[1]. In the present study, R package “WGCNA” was used to construct the gene co-expression network of the top 5000 genes with absolute median difference. Then clustered samples and the outlier samples are identified and removed. The scale-free topology network was constructed by setting the soft threshold at 6, R square = 0.85, and the minimum number of module genes as 30. Immune score was considered for phenotype files in WGCNA, and Pearson correlation analysis was used to identify the modules most relevant to immune score for subsequent analysis.

PPI network analysis

The protein-protein interaction (PPI) network was constructed using the online database STRING (https://cn.string-db.org/)[2]. followed by the Cytoscape software (version 3.9.1) to achieve visualization[3], and the “CytoHubba” plugin was used for modular analysis to identify the top 10 key genes.

Gene enrichment and function analysis

To verify the biological functions and pathways of the significant modules obtained by WGCNA analysis and VSIG4, we utilized The Database for Annotation, Visualization and Integrated Discovery (DAVID) database (https://david.ncifcrf.gov/) for Gene Ontology (GO) and Kyoto Encyclopedia of Genes and Genomes (KEGG) analysis[4], visualized by the R package “ggplot 2”.

GSVA

Gene Set Variation Analysis (GSVA) is an unsupervised gene enrichment strategy utilizing a new enrichment scoring algorithm to calculate the variations in pathway activity implied in the data[5]. The R package “GSVA” was used to explore the correlation of VSIG4 with 20 candidate functions covering a variety of immunity-related biological functions and pathways.

Association between VSIG4 and immune infiltration

CIBERSORT is an algorithm proposed by Newman et al. for characterizing cell composition from gene expression profiles of complex tissues[6]. We calculated the correlation between the expression level of VSIG4 in the database and the infiltration level of 22 major immune cells using the R package "CIBERSORT". To explore the depth of immune infiltration in CRC patients, VSIG4 expression profiles data in TCGA database and GSE14333 were grouped, and ssGSEA analysis was performed by R package "GSVA" to calculate the effect of VSIG4 expression level on common immune cells.

Survival analysis

By utilizing the R package “survival” and “survminer” to analyze clinical data from the TCGA and GEO databases for colorectal cancer, Kaplan-Meier plots were drawn to evaluate the patients’ overall survival of expression levels of VSIG4. A statistically significant difference was considered at P-value < 0.05.

Immunotherapy outcomes prediction

The Cancer Immunome Atlas (TCIA) Database (https://www.tcia.at/) characterized comprehensive immunogenomic analysis results for 20 solid cancers from TCGA data[7]. The immunophenotypic score (IPS) data of COAD and READ patients was extracted to predict the response to treatment with immune checkpoint (cytotoxic T lymphocyte antigen-4 (CTLA-4), programmed cell death protein-1/ programmed cell death-Ligand 1 (PD-1/PD-L1)) inhibitors.

Single-cell RNA-seq analysis

Single cell transcriptome data with ten pairs of primary colorectal cancer samples and matched normal mucosal samples were obtained from the GSE132465 database. Statistical analysis was performed using the "Seurat" package in R. Principal component analysis was performed for the top two thousand genes with the largest variables. T-distributed stochastic neighbor embedding (t-SNE) and uniform manifold approximation and projection (UMAP) algorithms are used to perform unsupervised clustering and unbiased visualization of cell subpopulations. The "FindAllMarkers" function was used to compare differences in gene expression between each cluster. Finally, each cluster is annotated manually by known markers.

**References**

1. Langfelder P, Horvath S. WGCNA: an R package for weighted correlation network analysis. BMC Bioinformatics. 2008; 9: 559.

2. Szklarczyk D, Franceschini A, Wyder S, Forslund K, Heller D, Huerta-Cepas J, et al. STRING v10: protein-protein interaction networks, integrated over the tree of life. Nucleic Acids Res. 2015; 43: D447-D52.

3. Shannon P, Markiel A, Ozier O, Baliga NS, Wang JT, Ramage D, et al. Cytoscape: a software environment for integrated models of biomolecular interaction networks. Genome Res. 2003; 13: 2498-504.

4. Huang DW, Sherman BT, Lempicki RA. Systematic and integrative analysis of large gene lists using DAVID bioinformatics resources. Nat Protoc. 2009; 4: 44-57.

5. Hänzelmann S, Castelo R, Guinney J. GSVA: gene set variation analysis for microarray and RNA-seq data. BMC Bioinformatics. 2013; 14: 7.

6. Newman AM, Liu CL, Green MR, Gentles AJ, Feng W, Xu Y, et al. Robust enumeration of cell subsets from tissue expression profiles. Nat Methods. 2015; 12: 453-7.

7. Charoentong P, Finotello F, Angelova M, Mayer C, Efremova M, Rieder D, et al. Pan-cancer Immunogenomic Analyses Reveal Genotype-Immunophenotype Relationships and Predictors of Response to Checkpoint Blockade. Cell Rep. 2017; 18: 248-62.


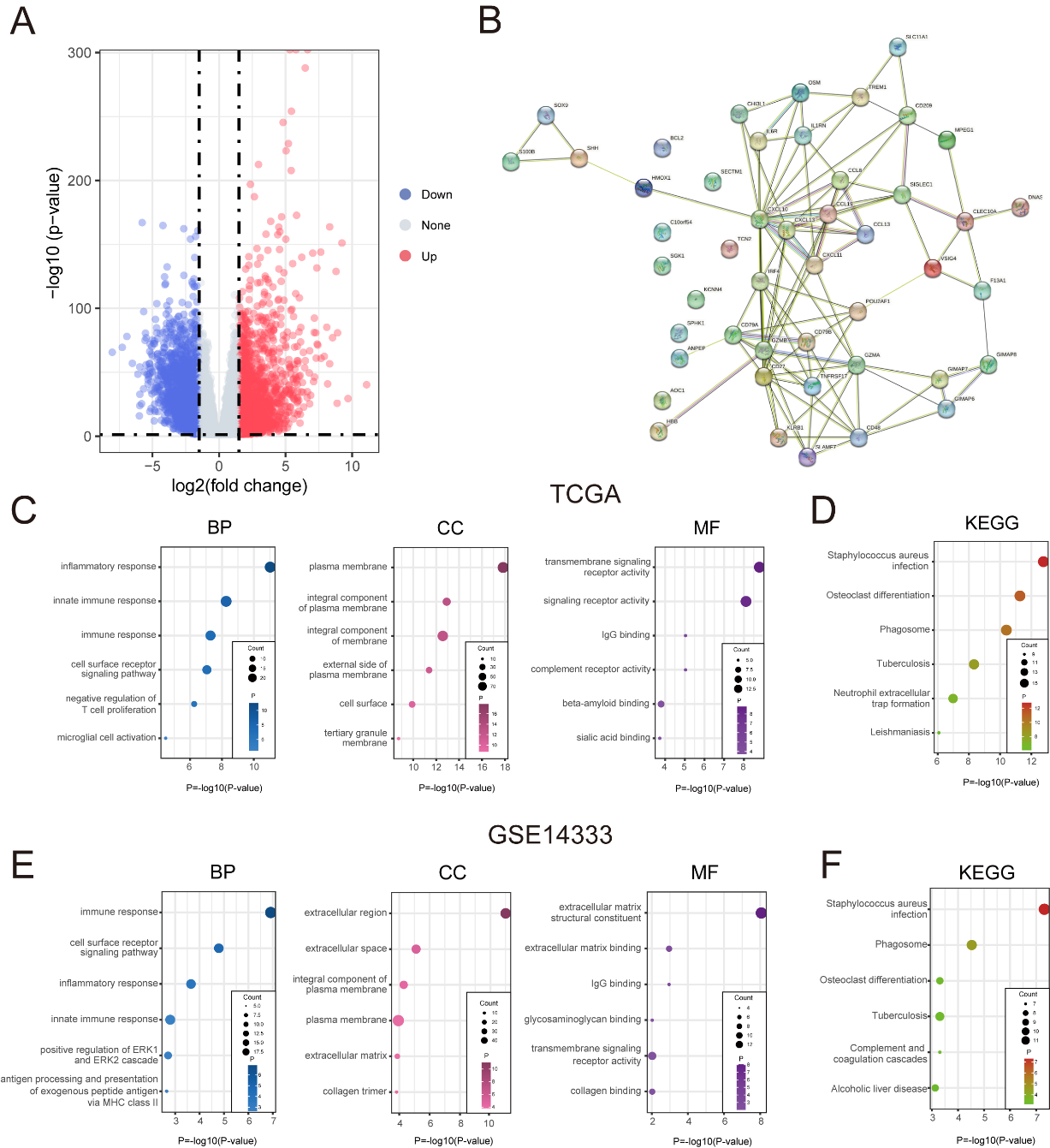


**Figure S1** (A) Volcano diagram of DEGs, threshold padj < 0.05, FoldChang > 1.5. (B) PPI networks of 47 key genes produced by STRING. Based on the TCGA database, GO (C) and KEGG (D) analyses revealed the relevant biological processes (BP), cell components (CC), molecular functions (MF), and pathways associated with VSIG4. Based on the GSE14333 database, GO (E) and KEGG (F) analyses revealed the associated biological processes (BP), cell components (CC), molecular functions (MF), and pathways associated with VSIG4.


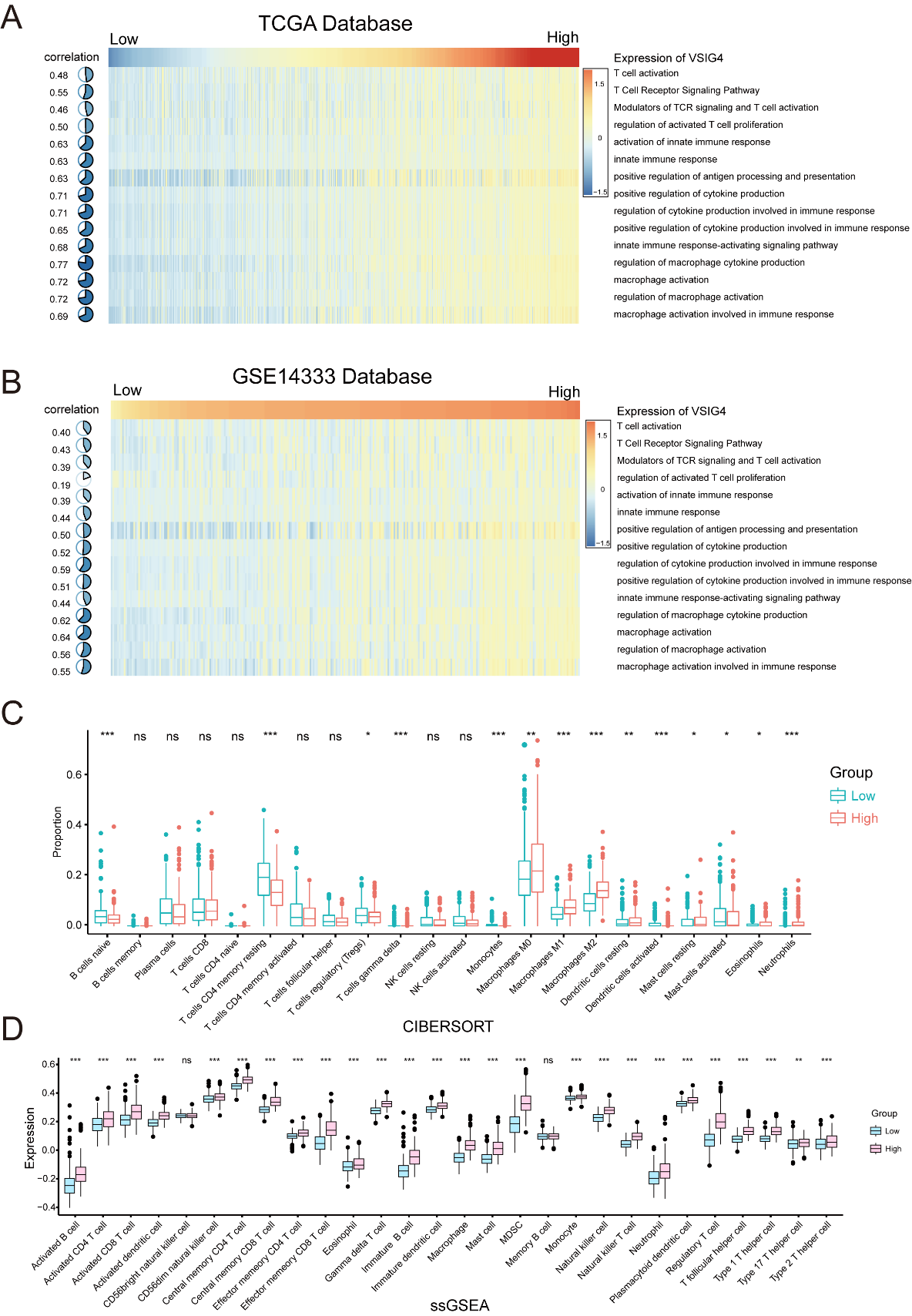


**Figure S2** Heatmap showing the enrichment scores of VSIG4 expression and immune function in the TCGA database (A) and the GSE14333 database (B) for each patient. The samples were arranged in ascending order of the expression of VSIG4. The pie chart on the left shows R-values of the correlation analysis. (C) The estimate of 22 common tumor-infiltrating lymphocytes between high VSIG4 expression group and low VSIG4 expression group. Red represents patients with high VSIG4 expression and blue represents patients with low VSIG4 expression. (d) Violin plot showing the relationship between VSIG4 expression levels and the abundances of different types of infiltrated immune cells by ssGSEA analysis. Red color represents patients with high VSIG4 expression and blue color represents patients with low VSIG4 expression.


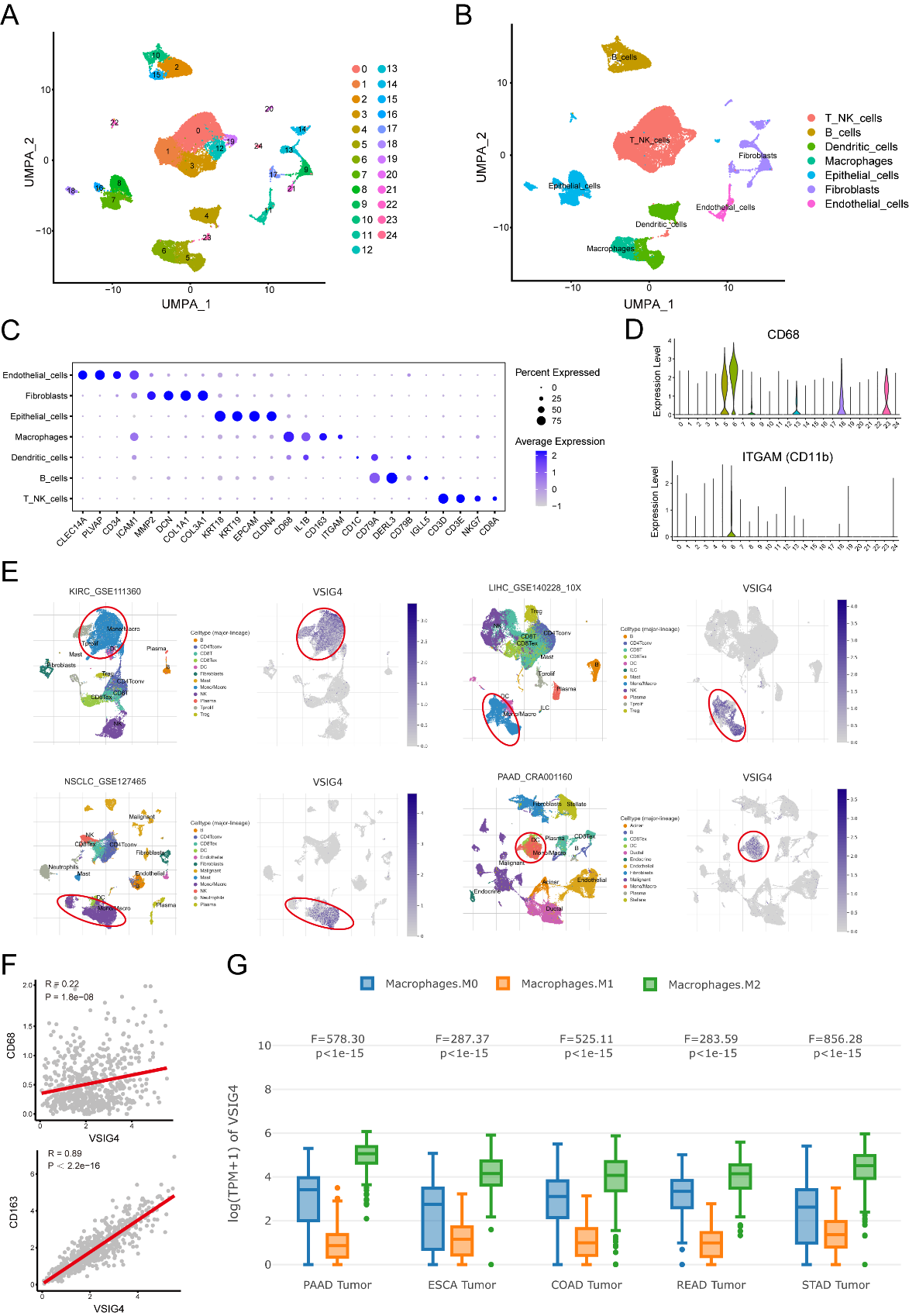


**Figure S3** (A) UMAP showing that ten pairs of primary colorectal cancer samples and matched normal mucosal samples were clustered into 25 cell clusters after single cell sequencing analysis. (B) UMAP showing 25 cell clusters divided into seven discrete categories according to the marked genes. (C) Bubble map showing the correlation between the seven types of cells and their respective marker genes. (D) Expression of macrophage marker genes ITGAM and CD68 in 25 cell clusters. (E) The pan-cancer single-cell sequencing dataset in the TISCH database (http://tisch.comp-genomics.org/) was used to analyze VSIG4 gene expression in KIRC, LIHC, NSCLC, and PAAD. (F) Correlation analysis between VSIG4 and CD68 and CD163 was based on RNA expression profiles in the TCGA database. (G) The GEPIA 2021 database was used to analyze the expression of VSIG4 in different macrophage subtypes in various tumors.


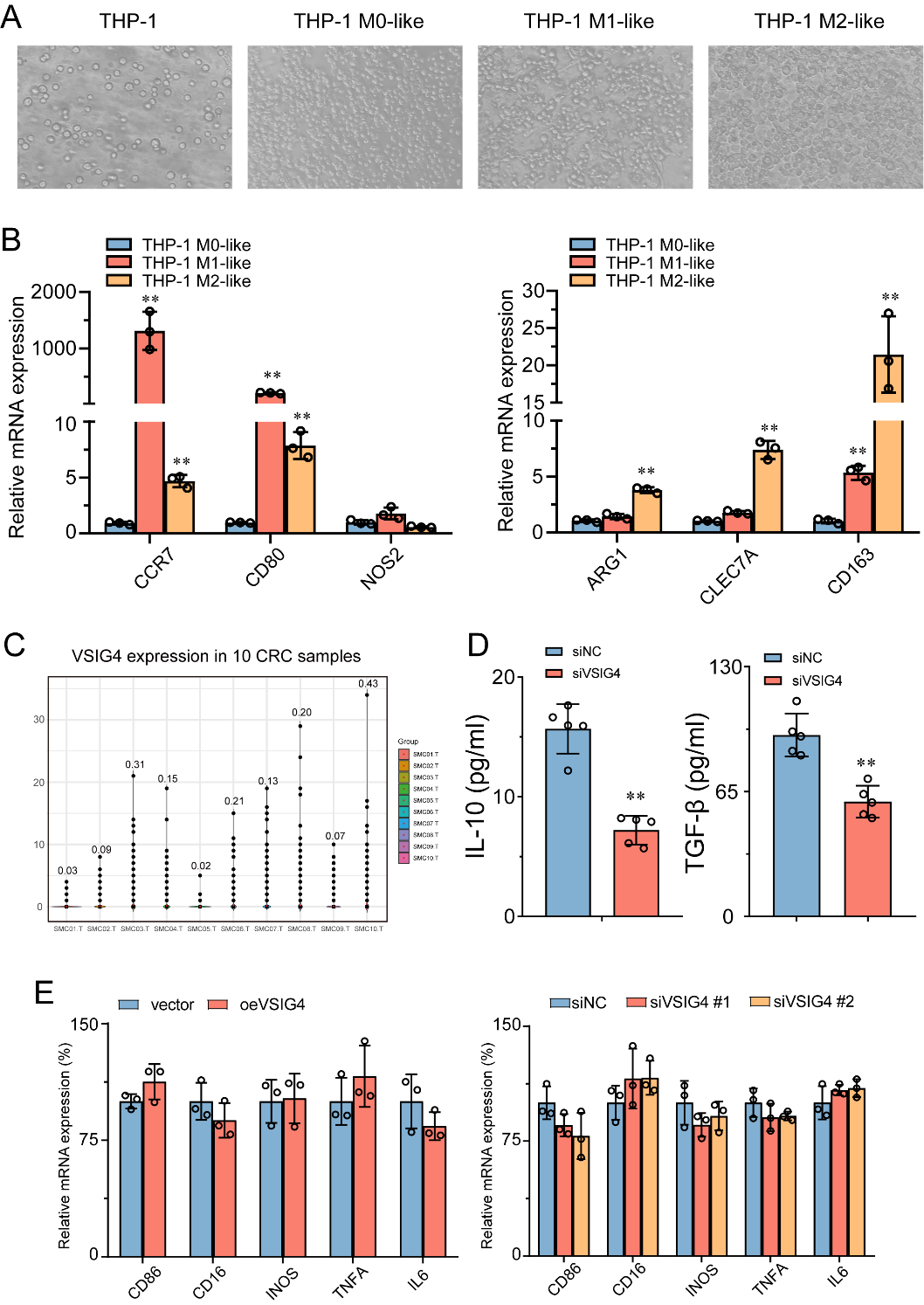


**Figure S4** (A) Microscopically revealing different morphologies of THP-1 monocytes as well as M0, M1 and M2 macrophages derived from THP-1 monocytes. (B) The mRNA levels of marker genes in M1 macrophages (CCR7, CD80, NOS2) and M2 macrophages (CD163, CLEC7A, ARG1) were detected by qRT-PCR. (C) Violin plot of VSIG4 expression in ten patients with primary colorectal cancer. (D) Secreted IL-10 and TGF-β in the supernatants of M2 macrophages detected by ELISA. (E) The mRNA levels of marker genes in M1 macrophages were detected by qRT-PCR.


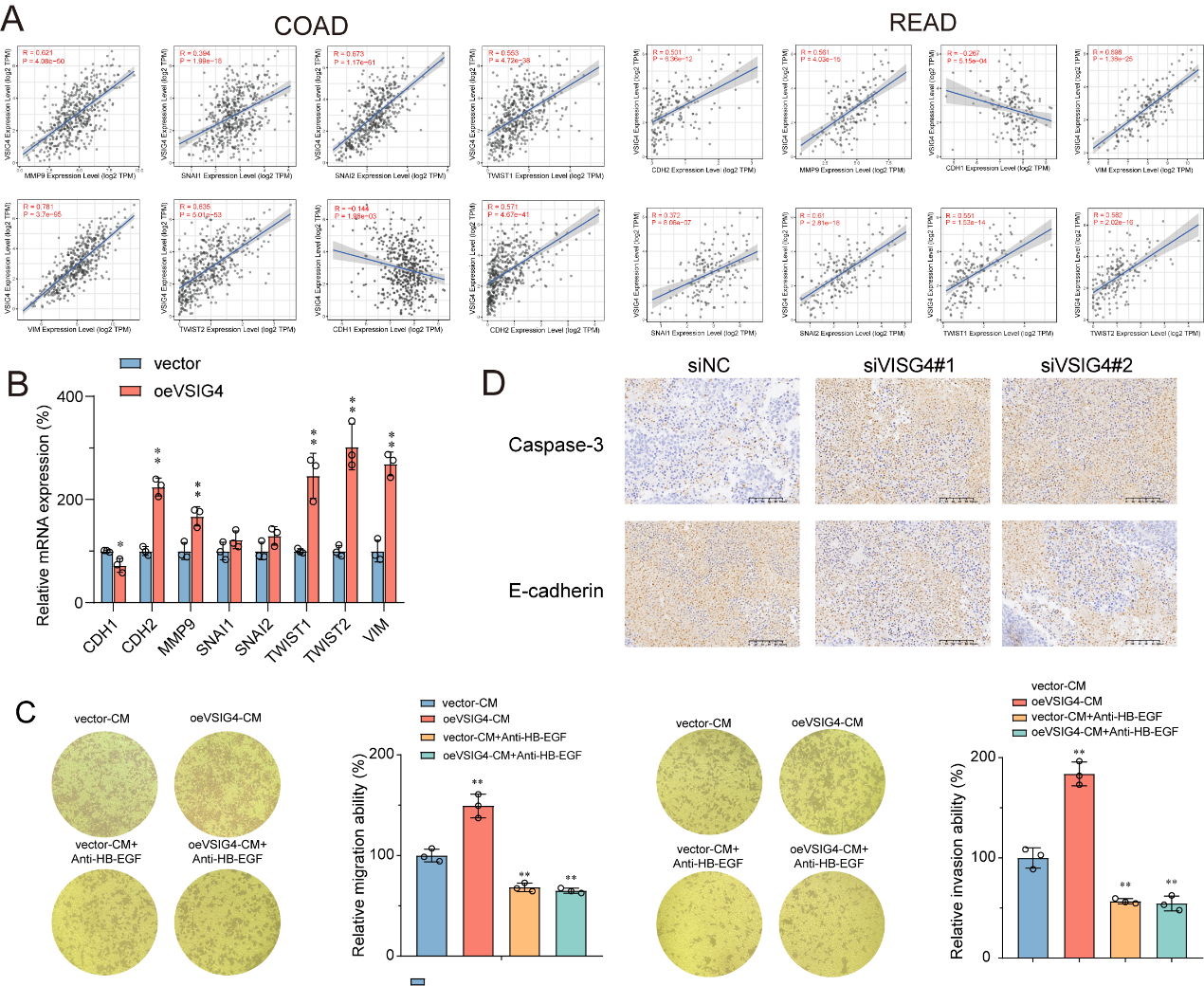


**Figure S5** (A) In COAD and READ cohorts, VSIG4 expression was positively correlated with EMT-related genes (CDH2(N-cadherin), MMP9, VIM, SNAI1, SNAI2, TWIST1, TWIST2) and negatively correlated with CDH1(E-Cadherin). (B) Following a 24-hour exposure of CRC cells to CM derived from M2-polarized macrophages, transcript levels of EMT-associated genes within tumor cells were systematically quantified via qRT-PCR. (C) Transwell assays of SW620 or LOVO cells with different treatments. Quantified data was shown on the right. (D) Immunohistochemical staining of Caspase-3 and E-cadherin specific antibodies. Scale, 100μm.


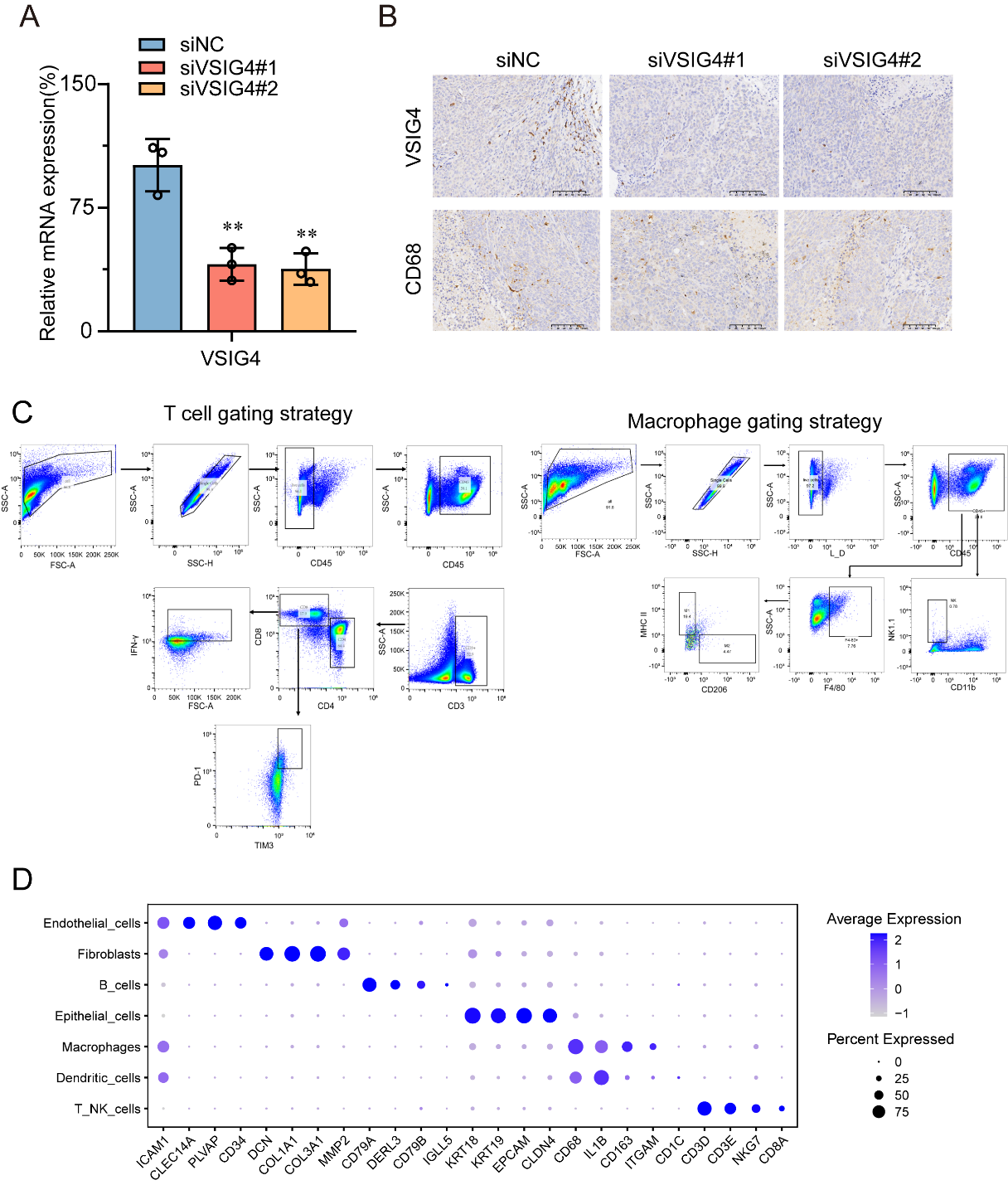


**Figure S6** (A) The mRNA levels of VSIG4 in TAMs was detected by qRT-PCR. (B) VSIG4 specific antibody immunohistochemical staining was used to detect the expression of VSIG4 in MC38 tumors. Scale, 100μm. (C) Total flow cytometric gating strategies for T cells and macrophages. (D) Bubble map showing the correlation between the seven types of cells and their respective marker genes.


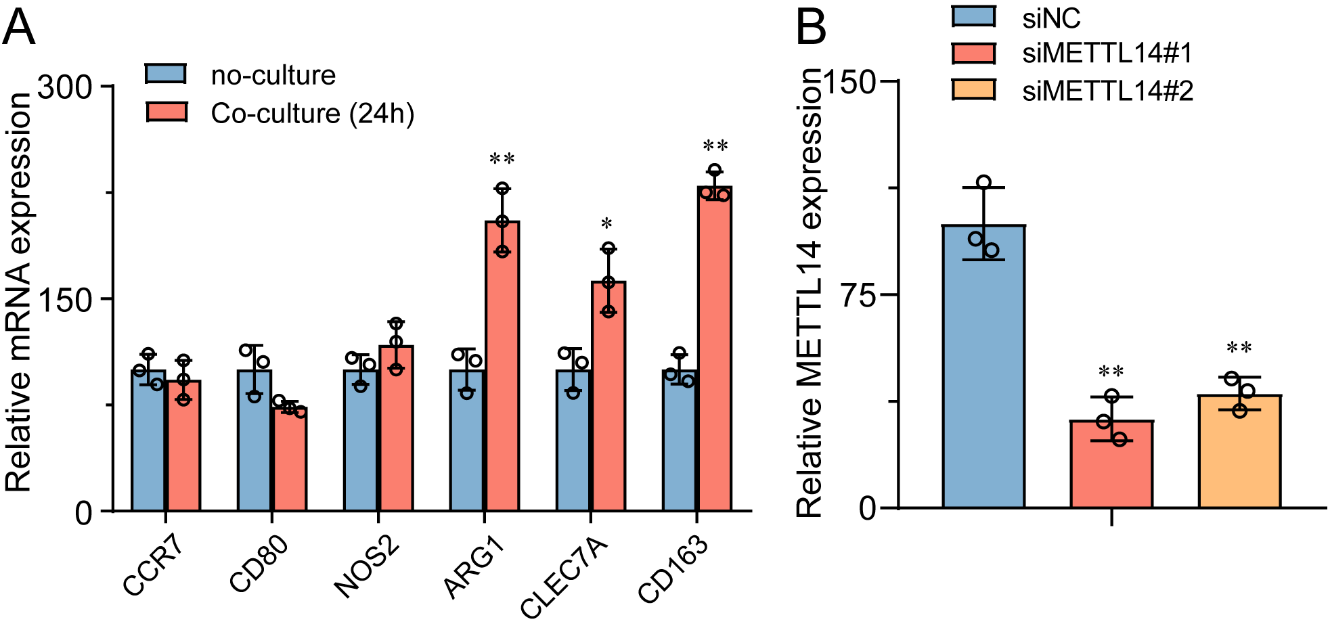


**Figure S7** (A) The mRNA levels of marker genes in M1 macrophages and M2 macrophages were detected by qRT-PCR. (B) The mRNA levels of METTL14 in PMA-activated THP-1 monocytes was detected by qRT-PCR.


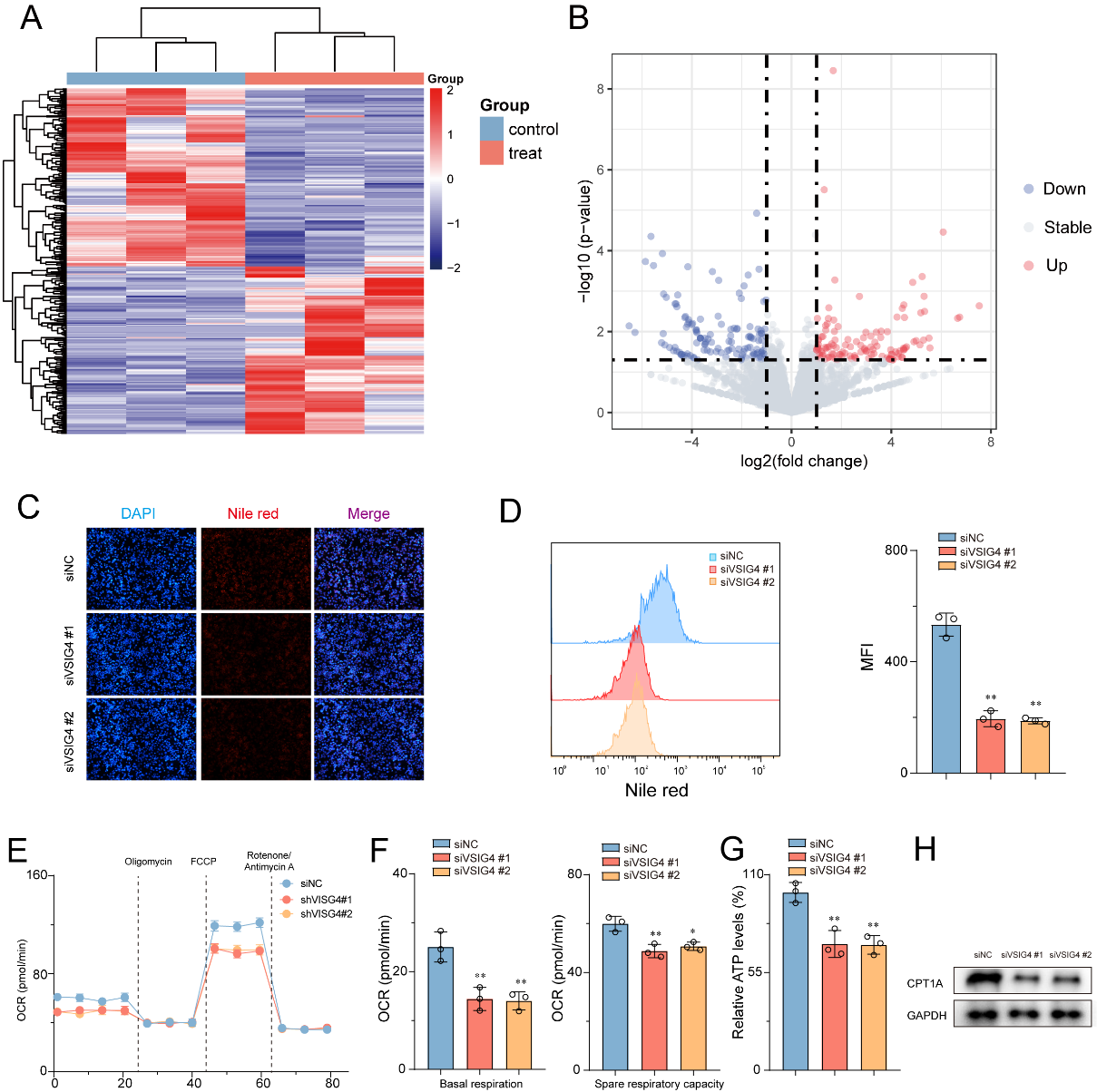


**Figure S8** (A) Heatmap showing the differential expression of genes in M2-polarized THP-1 monocytes overexpressed with VSIG4 and control M2-polarized THP-1 monocytes were analyzed by RNA-seq. (B) Volcano plot of differentially and non-differentially expressed genes in M2-polarized THP-1 monocytes overexpressed with VSIG4 and control M2-polarized THP-1 monocytes were analyzed by RNA-seq. (C) Representative images of Nile red fluorescence staining of M2-polarized THP-1 monocytes after VSIG4 knockdown. (D) The expression of Nile red in siVSIG4 M2-polarized THP-1 monocytes and control M2-polarized THP-1 monocytes was detected by FCM. (E) and (F) OCR and SRC of siVSIG4 M2-polarized THP-1 monocytes and control M2-polarized THP-1 monocytes were measured by Seahorse XFe 97 analyzer (n=3). (G) ATP production in siVSIG4 M2-polarized THP-1 monocytes and control M2-polarized THP-1 monocytes (n=3). (H) CPT1A protein expression in macrophages was detected by Western blot analysis.


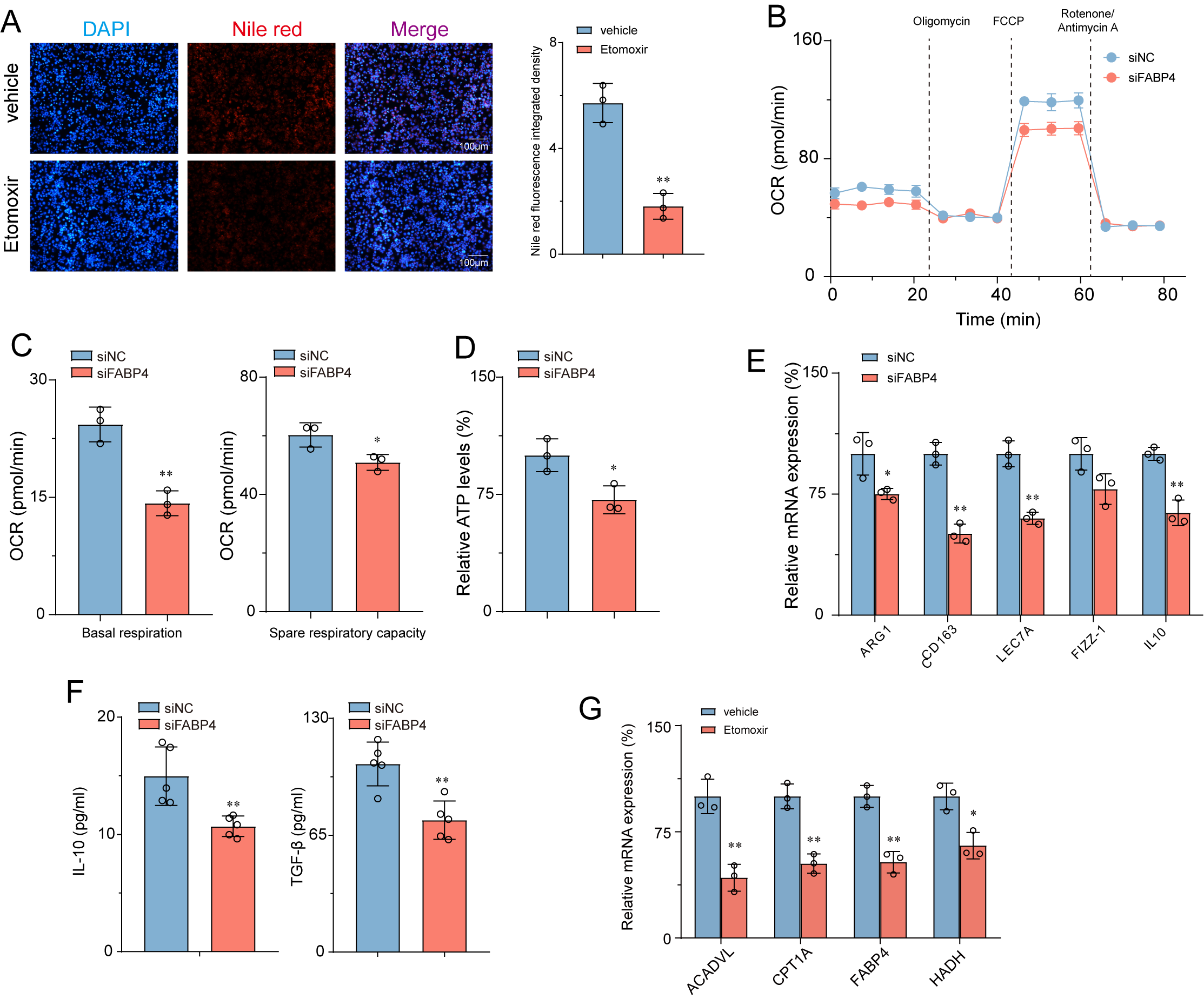


**Figure S9** (A) Representative images of Nile red fluorescence staining of M2-polarized THP-1 monocytes after Etomoxir treatment. (B) and (C) OCR and SRC of siFABP4 M2-polarized THP-1 monocytes and control M2-polarized THP-1 monocytes were measured by Seahorse XFe 97 analyzer (n=3). (D) ATP production in siFABP4 M2-polarized THP-1 monocytes and control M2-polarized THP-1 monocytes (n=3). (E) The mRNA levels of marker genes in M2 macrophages were detected by qRT-PCR. (F) Secreted IL-10 and TGF-β in the supernatants of M2 macrophages detected by ELISA. (G) qRT-PCR was used to detect mRNA levels of ACADVL, CPT1A, FABP4, HADH in M2 macrophages treated with Etomoxir.


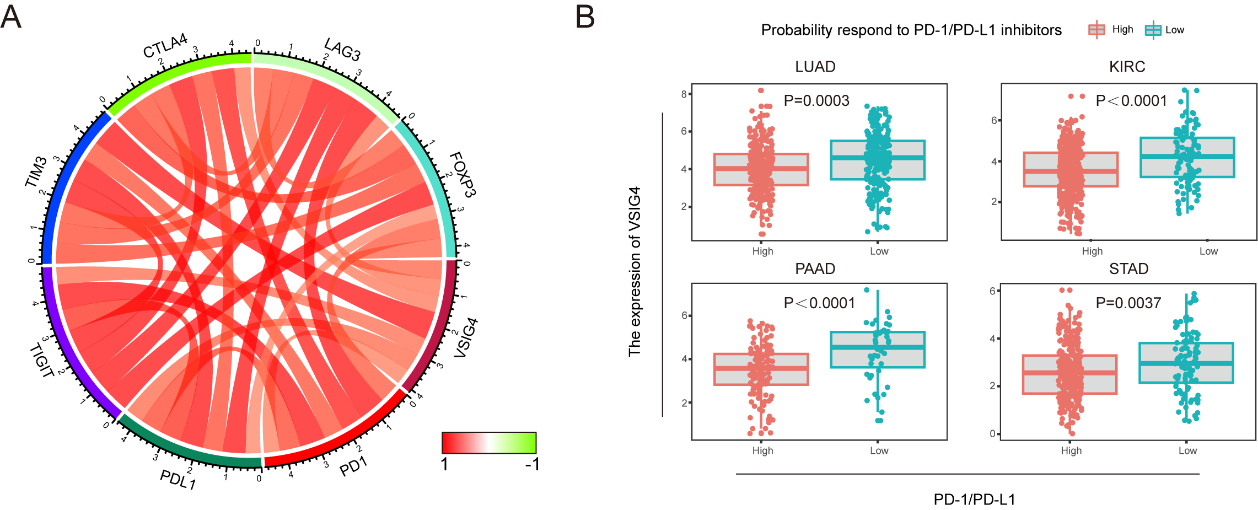


**Figure S10** (A) Circos plot of the correlation between VSIG4 and multiple inhibitory immune checkpoints, the darker the connection lines between plates or the thicker the lines, the higher the correlation. Red indicates a positive correlation, while green signifies a negative correlation. (B) The relationship between VSIG4 expression levels and the probability of response to immunotherapy (anti-PD-1 /PD-L1 treatment) across various cancer types.
